# Identification of aberrant innate and adaptive immunity based on changes in global gene expression in the blood of adults with autism spectrum disorder

**DOI:** 10.1186/s12974-021-02154-7

**Published:** 2021-04-30

**Authors:** Fumie Horiuchi, Yuta Yoshino, Hiroshi Kumon, Rie Hosokawa, Kiwamu Nakachi, Kentaro Kawabe, Jun-ichi Iga, Shu-ichi Ueno

**Affiliations:** Department of Neuropsychiatry, Molecules and Function, Ehime University Graduate School of Medicine, Shitsukawa, Toon, Ehime 791-0295 Japan

**Keywords:** Gene expression, RNA-sequencing, Autism spectrum disorder, WGCNA, Gene ontology, Innate immunity, Adaptive immunity

## Abstract

**Background:**

Autism spectrum disorder (ASD) is characterized as a neurodevelopmental disorder, and one of the main hypotheses regarding its cause is genetic factors. A previous meta-analysis of seven microarray studies and one RNA sequencing (RNA-seq) study using the blood of children with ASD identified dysregulation of gene expressions relevant to the immune system. In this study, we explored changes in global gene expression as the phenotype of ASD in the blood of adults with ASD.

**Methods:**

We recruited an RNA-seq cohort (ASD vs. control; *n* = 6 each) and a replication cohort (ASD vs. control; *n* = 19 each) and conducted RNA-seq to explore changes in global gene expression. We then subjected the significantly up- and downregulated genes to gene ontology (GO) and core analyses. Weighted gene correlation network analysis (WGCNA) was performed with all 11,617 genes detected in RNA-seq to identify the ASD-specific gene network.

**Results:**

In total, 117 significantly up- and 83 significantly downregulated genes were detected in the ASD compared with the control group, respectively (*p* < 0.05 and *q* < 0.05). GO analysis revealed that the aberrant innate and adaptive immunity were more obvious in the 117 upregulated than in the 83 downregulated genes. WGCNA with core analysis revealed that one module including many immune-related genes was associated with the natural killer cell signaling pathway. In the results for the replication cohort, significant changes with same trend found in RNA-seq data were confirmed for *MAFB* (*p* = 0.046), *RPSAP58* (*p* = 0.030), and *G2MK* (*p* = 0.004).

**Limitations:**

The sample size was relatively small in both the RNA-seq and replication cohorts. This study examined the mRNA expression level, so the interaction between mRNA and protein remains unclear. The expression changes between children and adults with ASD were not compared because only adults with ASD were targeted.

**Conclusions:**

The dysregulated gene expressions confirmed in the blood of adults with ASD were relevant to the dysfunction of innate and adaptive immunity. These findings may aid in understanding the pathogenesis of ASD.

**Supplementary Information:**

The online version contains supplementary material available at 10.1186/s12974-021-02154-7.

## Background

Autism spectrum disorder (ASD) is characterized as a neurodevelopmental disorder that has three main traits: stereotyped behaviors, deficits in communication, and diminished social skills. As of 2012, the median prevalence of ASD was reported to be 0.62% [1], and a diagnosis of ASD is more frequent in males than in females [2]. However, the current prevalence has risen for several reasons, including increased recognition, awareness, and changes in clinical practice or service availability [1]. Although multiple factors, environmental toxins and stressors, mitochondrial dysfunction, and impaired immune responses are involved in the pathogenesis of ASD [3], the mainstream hypothesis regarding its cause is the individual’s genetic background, such as single-nucleotide polymorphisms [4], rare genetic variants [5–7], and copy number variation [8–10]. As a nongenetic risk factor (in some cases, that interact with genetic factors), the most reliable and replicable association for ASD is advanced paternal age [11, 12]. The de novo mutations in sperm increase with paternal age [13–16], and most of the de novo mutations found in individuals with ASD come from the paternal origin [17, 18]. A growing body of evidence using postmortem brain studies has suggested the excessive production and increased density of microglia cells, especially in the dorsolateral prefrontal cortex [19], fronto-insular and visual cortex [20], cerebellum, midfrontal, and cingulate gyrus [21]. These findings are concordant with those from positron emission tomography studies [22]. At the morphology level, increased short-distance microglia–neuron interaction has been reported [23]. A transcriptome study revealed that these microglial changes were also found in gene expression levels, possibly induced by inflammatory cytokines [24]. Changes in inflammatory cytokines have also been found in the blood, including elevated pro-inflammatory cytokines such as TNF-α, interleukin (IL)-6, and IL-8, along with a decrease in anti-inflammatory cytokines such as TGF-β and IL-10 [25–27]. A meta-analysis of seven microarray studies using blood samples of children with ASD concluded that transcriptional cascades are typically elicited within circulating immune cells, contrary to the activated immune response in protein levels [28]. Recently, RNA sequencing (RNA-seq) has become a powerful method for investigating global gene expression as well as microarray data because it quantifies a large and dynamic range of expression levels with absolute rather than relative values. To our knowledge, only one transcriptome study has been conducted using RNA-seq of blood; the results revealed that immune dysregulation changes in gene expression levels were found based on genome-wide gene expression data using twin subjects with or without ASD [29]. Surprisingly, the one RNA-seq study and all seven microarray studies explored global gene expressions in the blood of only children with ASD. Considering that adults with ASD often experience more comorbidities as a result of facing social problems [30], gene expression among adults with ASD possibly differs from that among children with ASD because gene expressions generally change as a results of environmental factors such as smoking [31], alcohol consumption [32], and disease states. Given this background, the present study recruited adults with ASD and age- and sex-matched controls to investigate (1) global expression changes using RNA-seq of the blood, (2) the type of biological process to which differentially expressed genes (DEGs) belong based on the bio-computational method of gene ontology (GO) analysis, (3) ASD-specific gene networks using weighted gene correlation network analysis (WGCNA) with RNA-seq data, and (4) the expression profiles of selected genes in replication set using RNA-seq.

## Methods

### Demographic and clinical data

We recruited an RNA-seq cohort (ASD vs. control; *n* = 6 each) and a replication cohort (ASD vs. control; *n* = 19 each) and conducted RNA-seq to explore changes in global gene expression. The demographic and clinical data of the RNA-seq and replication cohorts are shown in Tables 1 and 2, respectively. A diagnosis of ASD was made according to the Diagnostic and Statistical Manual of Mental Disorders, Fifth Edition, by at least two expert psychiatrists on the basis of extensive clinical interviews and a review of medical records. The control subjects without psychiatric signs or a past history of mental disorders, and who were diagnosed as neurotypical normal based on clinical interviews by at least two expert psychiatrists, were recruited. The recruited individuals with ASD and controls were of unrelated Japanese origin and provided written informed consent before the study began in accordance with the ethics committee of Ehime University Graduate School of Medicine (No. 31-K8). The severity of ASD symptoms in the RNA-seq cohort were assessed by using the Autism Diagnostic Observation Schedule, Second Edition [33] or Autism Diagnostic Interview-Revised [34].

**Table 1:** Demographic and clinical data in the RNA-seq cohort

Sample	Age (years)	Sex	IQ	ADOS-2			ADI-R			RIN	Comorbidities
				Social domain	Communication domain	Repetitive behavior domain	Social domain	Communication domain	Repetitive, restricted and stereotyped interests and behaviors		
ASD 1	39	M	86	8	5	1				7.4	MDD
ASD 2	24	M	95	9	8	1				9.0	
ASD 3	22	M	< 35				25	16	3	8.7	ID
ASD 4	40	M	< 35				29	18	3	8.3	ID
ASD 5	39	F	37				28	14	6	8.5	ID
ASD 6	26	M	< 37				28	12	10	7.5	ID
Ct 1	39	M								9.1	
Ct 2	24	M								7.7	
Ct 3	22	M								7.8	
Ct 4	40	M								6.4	
Ct 5	39	F								6.6	
Ct 6	26	M								8.0	

*ADI*-*R* Autism Diagnostic Interview Revised, *ADOS*-*2* Autism Diagnostic Observation Schedule Second Edition, *ASD* autism spectrum disorder, *Ct* control, *F* female, *ID* intellectual disability, *IQ* intelligence quotient, *M* male, *MDD* major depressive disorder, *RIN* RNA integrity number

**Table 2:** Demographic and clinical data in the replication cohort

	ASD	Ct	*p* value
Number of samples	19	19	
Age (average ± S.D.)	31.5 ± 8.1	30.5 ± 8.1	0.76
Male:Female	12:7	12:7	1.0

*ASD* Autism Spectrum Disorder, *Ct* control

### RNA isolation and synthesis of complementary DNA

Blood was collected into PaxGene Blood RNA Systems tubes (BD, Tokyo, Japan), and RNA was isolated according to the manufacturer’s protocol. RNA concentration and quality were calculated by using the NanoDrop 1000 system (Thermo Fisher Scientific, Yokohama, Japan). RNA samples indicating a 260/280 ratio between 1.8 and 2.0 were assumed to be pure, and 1.0 μg of RNA was used to synthesize 40-μL reaction mixtures of complementary DNA (cDNA) using the High-Capacity cDNA Reverse Transcription Kit (Applied Biosystems, Foster City, CA, USA).

### Quantitative PCR

Reverse transcription quantitative PCR (RT-qPCR) was conducted to measure mRNA expression levels using the StepOnePlus Real-Time PCR System (Applied Biosystems). The Predesigned qPCR Assay used Hs.PT.58.15091972.g for MAFB, Hs.PT.58.38545657 for MARCKS, Hs.PT.56a.620140 for ALDH2, Hs.PT.58.39204572 for ETV7, Hs.PT.58.40751942 for BATF2, Hs.PT.58.4129391 for GNLY, Hs.PT.58.27150028.g for SCARNA17, Hs.PT.58.4951589 for CROCCP2, Hs.PT.58.915589 for RPSAP58, Hs.PT.58.20366823 for GZMK, Hs.PT.58.2433071 for TLR1, Hs.PT.58.1518186 for IL1B, Hs.PT.58.3630286 for TNFAIP6, and Hs.PT.39a.22214836 for GAPDH. RT-qPCR was conducted by using the PrimeTime Gene Expression Master Mix (Integrated DNA Technologies, Inc., Coralville, IA, USA) in a final volume of 10 μL. mRNA expression levels were measured in duplicate, and the same sample was used in each plate to remove errors between plates. The relative expression value was calculated by using Livak’s ΔΔCt method [35].

### RNA-sequencing

The RNA integrity number was measured with an Agilent 2100 Bioanalyzer (Agilent Technologies Inc., Santa Clara, CA, USA) and an Agilent RNA 6000 Nano Kit (Agilent Technologies Inc.), as shown in Table 1. Next, 200 ng total RNA from each sample was subjected to RNA-seq library preparation. Initially, globin mRNA and ribosomal RNA (rRNA) were removed using the NEBNext Globin & rRNA Depletion Kit (Human/Mouse/Rat) (New England Biolabs, Ipswich, MA, USA) and NEBNext Multiplex Oligos for Illumina (New England Biolabs). An RNA-seq library of each sample was prepared by using the Illumina TruSeq Stranded mRNA Sample Prep Kit (Illumina, Indianapolis, IN, USA) according to the manufacturer’s protocol. The quality of the average size (340–380 bp) was validated by using an Agilent 2100 Bioanalyzer and the Agilent DNA1000 kit (Agilent Technologies Inc.). The amount was determined using the qPCR method with the Kapa Library Quantification Kit (Illumina). The sequencing was done using the MiSeq Reagent kit V3 on a MiSeq system (Illumina) based on pair-end reads (75 bp) according to the manufacturer’s instructions. Sequencing was performed by running 150 cycles.

### Bioinformatic analysis of RNA sequencing data

Raw data files in FASTQ format were generated from the MiSeq system (Illumina). The reads were aligned to the reference genome (hg38) with TopHat software [36]. The expression levels (metric fragments per kilobase of transcript per million mapped reads [FPKM value]) of the known genes were estimated using Cufflinks [37]. The number of identified genes per group was calculated based on the average FPKM values ≥ 1.0 in each group. Genes including an FPKM value of 0 in either the ASD or control group were excluded. A total of 11,617 genes were analyzed according to the criteria. DEG analysis was performed using the edgeR package [38]. Statistical significance for the DEGs was set at *p* < 0.05 and *q* < 0.05. A volcano plot of DEGs was created using DEseq2 [39]. A heatmap based on significantly changed DEGs was generated by using heatmap.2.

### Weighted correlation network analysis

Weighted gene correlation networks based on the RNA-seq data were generated using the “WGCNA” R package [40]. Weighted gene correlation network analysis (WGCNA) was performed to identify gene modules and summarize clusters by the module eigengene or an intramodular hub gene in consideration of external traits according to the WGCNA tutorial (https://horvath.genetics.ucla.edu/html/CoexpressionNetwork/Rpackages/WGCNA/). A “signed network adjacency” was applied to construct a network from the RNA-seq data (12 subjects: *n* = 6 each in the ASD and control groups) by setting a soft thresholding power of “6,” which was determined according to the result of scale-free topology and mean connectivity (Figure S1). The adjacency matrix was converted into a “topological overlap matrix (TOM)” to minimize the effects of noise and spurious associations. The “dynamicTreeCut” R package was used to detect groups of highly correlated genes. The corresponding dissimilarity was calculated as “1 – TOM.” Then, the modules were merged by setting a cut height value of 0.25′, a deep split of 2′, and a minimum module size of 30′ (Figure S2). For the created modules, the module eigengene was calculated by using the “ModuleEigengenes” function. Correlation analysis between a module and each parameter (trait [ASD or control subjects], age, and sex) was performed using the “corPvalueStudent” function, with statistical significance set at *p* < 0.05. The genes of the interested module were then subjected to functional annotation and core analysis.

### Functional annotation

The gene ontology (GO) of biological process (BP), molecular function (MF), and cellular component (CC) were conducted by using the ClueGO plugin [41] in the Cytoscape program (ver. 3.8.0). Statistical significance was set at *p* < 0.05 and corrected by suing the Benjamini–Hochberg method. Graphical networks of significant GO terms were created by using GO term fusion based on the following criteria: visual style = Groups; GO Term/Pathway Network Connectivity = Medium (kappa score = 0.50).

### Core analysis (canonical pathway, diseases, and functions)

Ingenuity Pathway Analysis software (Qiagen, Valencia, CA, USA) was used to consider a module for the functional enrichment of target genes and decipher their role in canonical and disease pathways by using Fisher’s exact test, with the p value threshold set at < 0.05.

### Statistical analysis

SPSS 22.0 software (IBM Japan, Tokyo, Japan) was used for the statistical analysis. The assessment of a normal distribution was considered by using the Shapiro–Wilk test. Average differences in age and mRNA levels were assessed by using Student’s *t* test or the Mann–Whitney *U* test. Differences in sex were analyzed with Fisher’s exact test. Correlations between gene expressions and covariates were conducted by suing the Pearson correlation coefficient or Spearman’s rank correlation coefficient. The average differences of mRNA expression in qPCR by sex were tested by using Student’s *t* test or the Mann–Whitney *U* test. Statistical significance was defined at the 95% level (*p* = 0.05).

## Results

### Demographic data

No significant differences were found in the demographic data between the ASD and control subjects in the RNA-seq cohort (age: *p* = 1.0, sex: *p* = 1.0) or the replication cohort (age: *p* = 0.76, sex: *p* = 1.0).

### RNA-seq

**Fig. 1:**
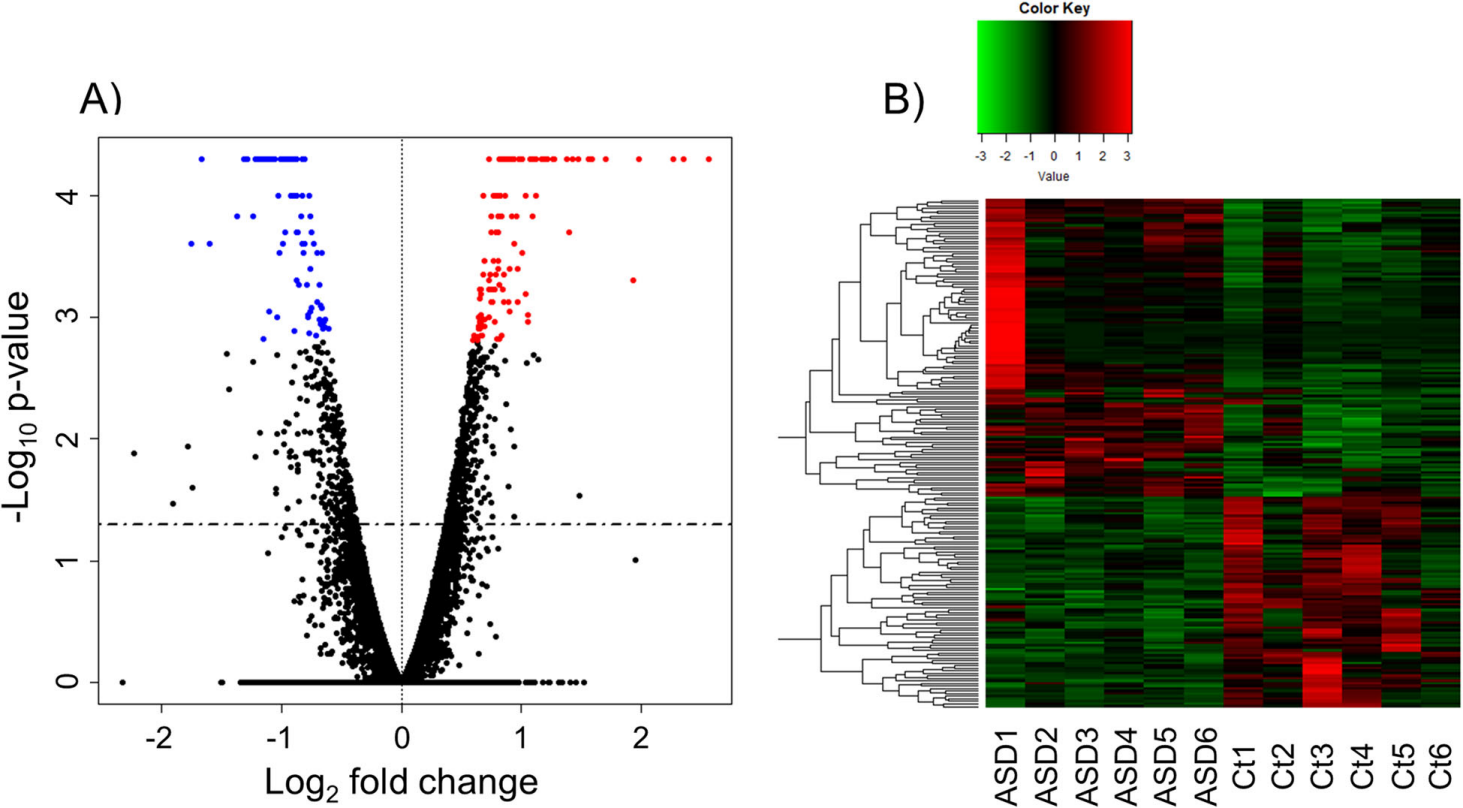
RNA sequencing data in a volcano plot and heatmap. **a** FPKM values of 11,617 detected genes were plotted in a volcano plot. The *y*-axis corresponds to the significance level represented with log_10_P value, and the *x*-axis displays the log_2_ (FC) value. The red dots represent the significantly (*p* < 0.05 and *q* < 0.05) upregulated genes in the ASD group; the blue dots represent the significantly (*p* < 0.05 and *q* < 0.05) downregulated genes in the ASD group. The dotted horizontal line means *p* = 0.05 [–log_10_ (1.30)]. **b** High expression genes are shown in red on the map; low expression genes are shown in green. The 117 up- and 83 downregulated DEGs were used to create the heatmap. *ASD* autism spectrum disorder, *Ct* control, *DEGs* differentially expressed genes, *FC* fold change

A total of 11,617 genes were used for subsequent analysis under stringent criteria, as mentioned in the “Methods” section; these genes are depicted in the volcano plot in Fig. 1a. Of these genes, 117 significantly upregulated and 83 significantly downregulated genes were detected in the ASD compared with the control group (*p* < 0.05 and *q* < 0.05). A heatmap of significantly changed DEGs is shown in Fig. 1b. The high fold-change genes are shown in Table 3, and all significantly changed genes are shown in Table S1.

**Table 3:** High fold-change genes among the significantly up- and downregulated genes

Gene symbol	Locus	ASD FPKM average value	Ct FPKM average value	Fold change	*p* value	*q* value
Upregulated genes top 10-fold change						
*ETV7*	chr6:36354220-36387800	12.8652	2.18622	5.884678	5.00E-05	0.004845
*BATF2*	chr11:64987944-64997045	13.3396	2.61341	5.104289	5.00E-05	0.004845
*SERPING1*	chr11:57597553-57614853	24.502	5.10407	4.800483	5.00E-05	0.004845
*CD274*	chr9:5450502-5470567	10.0294	2.53744	3.952566	5.00E-05	0.004845
*FCGR1A*	chr1:149782688-149812373	16.4805	4.31197	3.822035	0.0005	0.024958
*WARS*	chr14:100333787-100376343	125.775	38.7785	3.243421	5.00E-05	0.004845
*CASP5*	chr11:104994239-105023168	18.9458	6.30641	3.004213	5.00E-05	0.004845
*PI3*	chr20:45174898-45176544	189.799	64.3728	2.948435	5.00E-05	0.004845
*FCGR1B*	chr1:121087344-121097161	29.3301	10.5953	2.768218	5.00E-05	0.004845
*TMEM176B*	chr7:150791287-150805120	54.7088	20.312	2.693423	5.00E-05	0.004845
Downregulated genes top 10-fold change						
*RPSAP58*	chr19:23763013-23828117	3.19528	10.7714	0.296645	0.00025	0.014708
*GZMK*	chr5:55024278-55034132	6.6229	20.9275	0.316469	5.00E-05	0.004845
*KLRB1*	chr12:9595273-9607901	7.52223	22.75	0.330647	0.00025	0.014708
*GZMA*	chr5:55102645-55110252	9.13986	23.5862	0.387509	0.00015	0.010189
*DUSP2*	chr2:96143168-96145440	6.79868	16.8911	0.402501	5.00E-05	0.004845
*GNLY*	chr2:85694290-85698851	143.126	348.618	0.410553	5.00E-05	0.004845
*A2M-AS1*	chr12:9065176-9115962	7.10154	16.7187	0.424766	0.00015	0.010189
*TOMM7*	chr7:22812632-22822852	17.1637	39.7824	0.43144	5.00E-05	0.004845
*LINC00612*	chr12:9055588-9065070	5.26144	12.134	0.433611	5.00E-05	0.004845
*SNORD3A*	chr17:19188015-19188232	261.405	592.336	0.441312	5.00E-05	0.004845

### Functional annotation and core analysis in the RNA-seq data

**Fig. 2:**
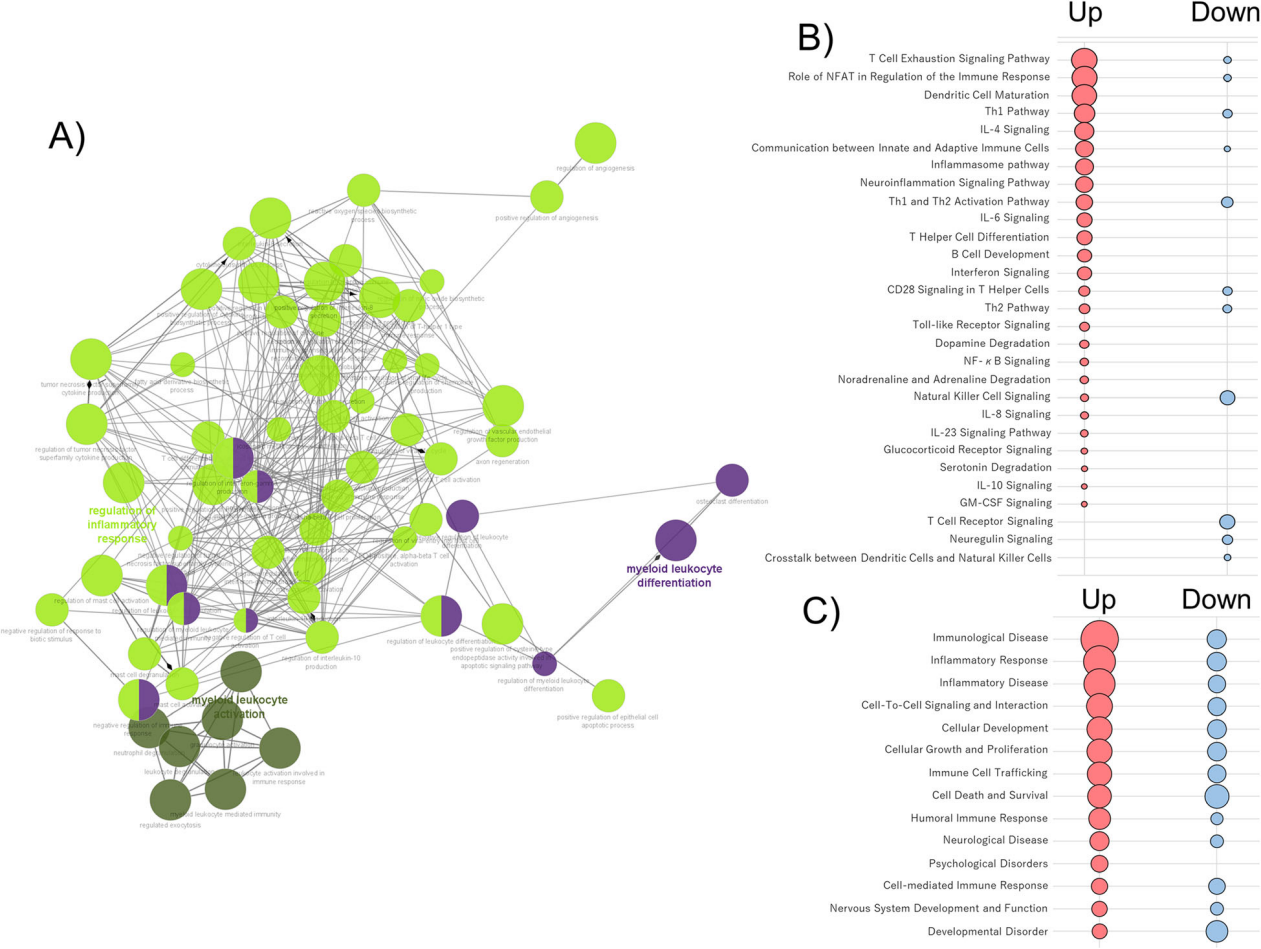
Immune-related BP results and core analysis based on RNA-sequencing data. **a** The network was constructed by immune-related BP terms based on 117 upregulated genes (*p* < 0.05 and *q* < 0.05), which were revealed by using ClueGo in the Cytoscape program. Canonical pathways, disease, and function were analyzed using 117 up- and 83 downregulated genes (*p* < 0.05 and *q* < 0.05). Significant results for the canonical pathway (**b**) and disease and function (**c**) are shown in red (up) and blue (down) circles. Spot/circle size is the function of –log(base=10) of Fisher’s exact test enrichment p value. *BP* biological process

All 117 upregulated genes were subjected to analysis because the fold change of 117 genes was more than 1.5. As a result, 90 BP terms reached significant levels (Table S2). Of those BP terms, immune-related BP terms were abundant and had functionally interacted with each other (Fig. 2a). In terms of CC and MF terms, 18 CC and four MF terms reached statistical significance. The terms of ficolin-1-rich granule lumen (*p* = 0.00045) and ether hydrolase activity (*p* = 0.00012) were most significant in CC and MF, respectively. For the same reason, all 83 downregulated genes were used to conduct functional annotation. Eight BP terms, including immune response, and six CC terms were found to be significant terms, but no significant result was found in MF (Table S3). When considering canonical pathways, several inflammatory pathways and immune responses were relevant to both up- and downregulated genes. Those immune-related pathways and responses were more abundant in upregulated than in downregulated genes (Fig. 2b). This trend was similar to the results of diseases and functions. In addition, neurological disease, nervous system development and function, and development disorder overlapped between up- and downregulated genes, but psychological disorders were found only in upregulated genes (Fig. 2c).

### WGCNA

**Fig. 3:**
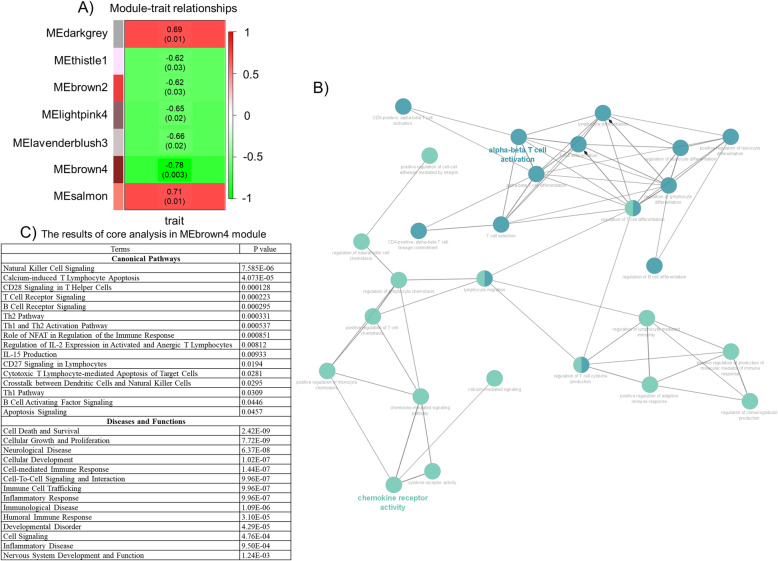
The results of WGCNA, gene ontology, and core analysis based on the enriched immune-related genes (MEbrown4) module. **a** The figure represents only the significant module eigengene correlated with phenotypic traits (ASD or control). Each row represents the module eigengene or ME (the correlation matrix of the module and sample, labeled by color). **b** The network was constructed by immune-related BP terms based on 479 genes involved in MEbrown4, which were revealed by using ClueGo in the Cytoscape program. **c** Significant results for the canonical pathway and disease and function are based on 479 genes involved in MEbrown4. *ASD* autism spectrum disorder

WGCNA analysis identified 43 co-expression gene network modules (Figure S3), among which, MEdarkgrey (*r* = 0.69, *p* = 0.01, *n* = 740), MEthistle1 (*r* = − 0.62, *p* = 0.03, *n* = 75), MEbrown2 (*r* = − 0.62, *p* = 0.03, *n* = 44), MElightpink4 (*r* = − 0.65, *p* = 0.02, *n* = 77), MElavenderblush3 (*r* = − 0.66, *p* = 0.02, *n* = 74), MEbrown4 (*r* = − 0.78, *p* = 0.003, *n* = 479), and MEsalmon (*r* = 0.71, *p* = 0.01, *n* = 188) were associated with traits (ASD or control subjects), as shown in Fig. 3a. Subsequently, we focused on the MEbrown4 module, which was the most significant (479 genes, as shown in Table S4).

### Functional annotation and core analysis in the MEbrown4 module

All 479 genes were subjected to analysis. As a result, 78 BP terms reached significant levels (Table S5). Numerous immune-related BP terms functionally interacted with each other (Fig. 3b). Moreover, 11 MF terms reached significant levels, with core promoter sequence-specific DNA binding being the most significant (*p* = 0.022). No significant CC terms were found. Core analysis (canonical pathways, diseases, and functions) also revealed a relationship between the MEbrown4 module and immune-related terms (Fig. 3c).

### qPCR validation for RNA-seq data and replication set

**Fig. 4:**
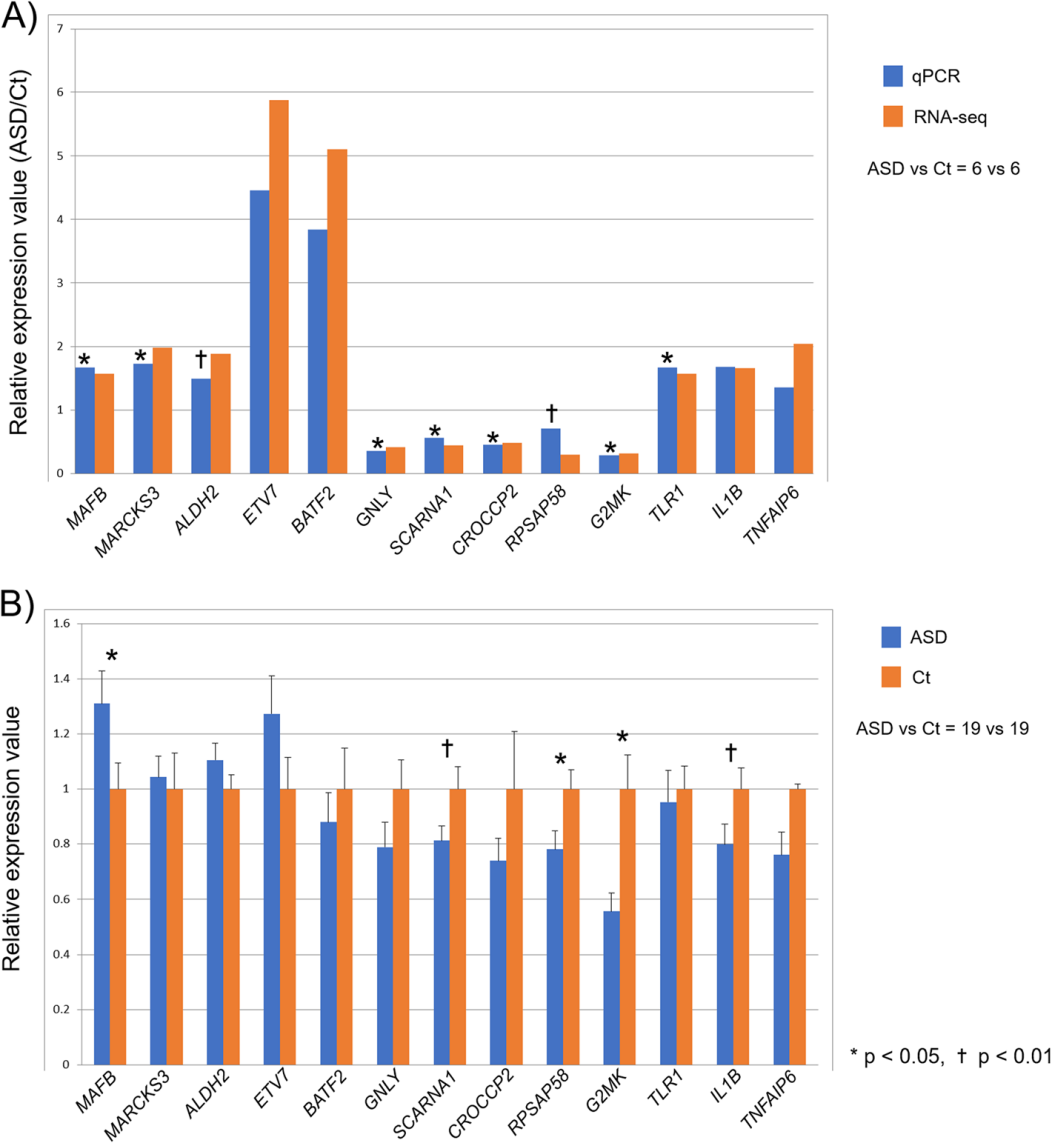
Validation qPCR in the RNA-seq and replication cohorts. **a** The *y*-axis represents the ratio of the relative expression value (ASD/Ct) in qPCR and RNA-seq, and the *x*-axis represents the gene names selected by the results of RNA-seq. This experiment was done in the RNA-seq cohort. **b** The *y*-axis represents the ratio of relative expression values of ASD and control subjects, and the *x*-axis represents the gene names selected by the results of RNA-seq. This experiment was done in the replication cohort. *p < 0.05; †*p* < 0.01. *ASD* autism spectrum disorder, *Ct* control

The same samples used in RNA-seq (ASD vs. control subjects = 6 vs. 6) were tested in a validation qPCR experiment. We picked up three most significant (*p* = 5.00E-05) and two highest fold change genes about up and downregulated genes, respectively. Additionally, three genes associated with the immune system (*TLR1*, *IL1B*, and *TNFAIP6*) which were significantly upregulated in RNA-seq result were measured. The trends in expression and fold changes of the qPCR results were similar to the RNA-seq data for all 13 genes (Fig. 4a). Significant changes were found for *MAFB* (*p* = 0.002), *MARCKS3* (*p* = 0.049), *GNLY* (*p* = 0.014), *SCARNA1* (*p* = 0.005), *CROCCP2* (*p* < 0.001), *G2MK* (*p* = 0.013), and *TLR1* (*p* = 0.041). There were no significant changes in *ALDH2* (*p* = 0.065), *ETV7* (*p* = 1.0), *BATF2* (*p* = 0.59), *RPSAP58* (*p* = 0.086), *IL1B* (*p* = 0.13), or *TNFAIP6* (*p* = 1.0).

In terms of the replication set, significant changes with the same trends found in the RNA-seq data were confirmed for *MAFB* (*p* = 0.046), *RPSAP58* (*p* = 0.030), and *G2MK* (*p* = 0.004), as shown in Fig. 4b. Similar change trends were found for *SCARNA1* (*p* = 0.080), whereas the opposite was found for *IL1B* (*p* = 0.066). No significant changes were observed for *MARCKS3* (*p* = 0.77), *ALDH2* (*p* = 0.20), *ETV7* (*p* = 0.13), *BATF2* (*p* = 0.73), *GNLY* (*p* = 0.18), *CROCCP2* (*p* = 0.75), *TLR1* (*p* = 0.56), or *TNFAIP6* (*p* = 0.32).

Next, the mRNA expression levels measured by using qPCR in the replication cohort were evaluated in terms of their association with covariates such as age and sex. No changes were associated with covariates except for *ALDH2* (age in ASD, *p* = 0.019, *r* = 0.534), *RPSAP58* (sex in ASD, *p* = 0.031: male vs. female = 1.14 ± 0.44 vs. 1.63 ± 0.43), and *TNFAIP6* (sex in ASD, *p* = 0.008: male vs female = 1.14 ± 0.56 vs. 2.11 ± 0.0.55), as shown in Table S6.

## Discussion

To our knowledge, this is the first study to conduct RNA-seq using the blood of adults with ASD and unrelated control subjects. Both up- and downregulated DEGs were associated with the immune system based on the results of bio-computational analyses. Additionally, WGCNA revealed that one module (gene network) was significantly correlated with ASD pathogenesis through numerous immune-related genes.

Most of the selected genes were successfully validated in the qPCR experiment within the same samples used in RNA-seq. However, only a few had significant and identical direction changes in the replication set; the other genes showed no significant changes, although the directions were almost the same. In addition to ASD pathogenesis, individual backgrounds (e.g., lifetime event. parenting, comorbidities) could affect gene expression changes [42]. In this regard, many samples are needed to verify this point.

In addition, a larger number of immune-related BP terms was confirmed when subjecting the 117 upregulated DEGs to GO analysis compared with the 83 downregulated DEGs, and these genes also showed higher interaction with each other. Especially, many BP terms were related to innate immunity (e.g., myeloid leukocyte mediated immunity, neutrophil degranulation, mast cell degranulation), which is one of the two main types of immunity in vertebrates (the other is the adaptive immune system). Children with ASD have specific reactions toward benign factors such as common illnesses and environmental challenges [43]. These children often suffer from inflammation of the gastrointestinal mucosa and nodular hyperplasia, and in turn, present with innate immune abnormalities [14, 44]. Numerous studies have reported immune dysregulation, which is reported as increased levels of pro-inflammatory cytokines secreted by peripheral blood mononuclear cells [25, 45, 46]. Indeed, the dysregulation of cytokine production was revealed in upregulated DEGs from both BP terms and the canonical pathway. On the other hand, the canonical pathway revealed that the upregulated DEGs were involved in the adaptive immune system (e.g., Th1 pathway, Th2 pathway, Th1 and Th2 activation pathway), in addition to BP terms such as CD4-positive, alpha-beta T cell activation, positive regulation of T-helper 1 type immune response, and T cell differentiation involved in the immune response. Replicative findings have shown atypical adaptive T cell responses in ASD subjects [47]. One possible reason for this is that the dysregulation of Th1 and Th2 immune responses induces the elevated pro-inflammatory cytokine TNF-α in ASD subjects. Interestingly, Ashwood et al. reported that increased TNF-α was associated with stereotypical behaviors in ASD subjects [47]. In fact, the gene expression changes found in the replication cohort were affected by age and sex among ASD subjects. This suggests that the expression change of immune-related genes could be affected by ASD symptoms, age, and sex as well as ASD pathogenesis. Compared with elevated pro-inflammatory cytokines such as TNF-α (Th1), IL-6 (Th2), GMCSF, and IL-8 (multiple immune cells), anti-inflammatory cytokines (e.g., TGF-β, IL-10) are commonly decreased in the blood [25–27, 48]. Collectively, our results suggest that both innate and adaptive immunity are dysregulated in adults with ASD and possibly induce increased pro-inflammatory and decreased anti-inflammatory cytokines.

Moreover, WGCNA identified one module (MEbrown4, 479 genes) that is inversely correlated with ASD. These genes are functionally highly interacted with each other and relevant to the immune system, as verified by BP terms and core analyses. This result suggests that dysregulated immune-related genes are possibly affected by ASD pathogenesis together, not individually, which is revealed by WGCNA. Central nervous system (CNS) activity may generally impact immunological functions via neurotransmitters and glucocorticoids. Conversely, pro-inflammatory cytokines, monocytes, macrophages, and T or B lymphocytes from the peripheral immune system act on the CNS through microglial cell activation [49, 50]. Interestingly, the most significant canonical pathway was the “natural killer (NK) cell signaling” pathway, which is responsible for innate immunity. The association between ASD and NK cells has been frequently discussed [51]. A recent study suggested that immune dysregulation was implicated in the higher expression of NK cytotoxicity genes in the blood of children with ASD [52]. Of the seven genes that were highly annotated for NK activity, four (*SPON2*, *IL2RB*, *PRF1*, and *CX3CR1*) were found in this module. The authors also investigated CD56^+^ NK cells under both stimulated and unstimulated situations and found higher resting but reduced cytolytic activity compared with the control subjects. A more recent study involving 104 children with ASD indicated significantly lower levels of CD57^+^ NK cells in children with ASD despite having a normal level of CD56^+^ NK cells [53]. In essence, the specific gene network revealed by WGCNA is implicated in the lower activity of NK cells, even in adults with ASD.

## Limitations

This study had several limitations. First, four of six ASD subjects in the RNA-seq cohort have intellectual disability, and could not take Autism Diagnostic Observation Schedule. We did not investigate how the effect of ASD severity impacts global gene expressions. Second, the sample size was relatively small in both the RNA-seq cohort (ASD vs. control; *n* = 6 each) and the replication cohort (ASD vs. control; *n* = 19 each). In this regard, a type I error may be present; however, most of the significant genes found in RNA-seq were successfully validated in the qPCR experiment. Third, most preceding studies have verified the dysregulation of the immune system from the perspective of protein levels. The present study explored the mRNA expression level, so the interaction between mRNA and protein remains unclear. Lastly, we targeted only adults with ASD. Basically, the three main traits of ASD are found at birth and last a lifetime [54]. From this viewpoint, the pathogenesis of ASD lasts from birth to adulthood, but several factors, such as comorbidities, lifestyle, and drugs, could affect gene expression. In the future, expression changes in both mRNA and protein levels need to be investigated in larger cohorts of children and adults with ASD.

## Conclusion

The dysregulated gene expressions confirmed in the blood of adults with ASD were relevant to the dysfunction of innate and adaptive immunity, and those possibly induce the increased pro-inflammatory and decreased anti-inflammatory cytokines. These findings may aid in understanding the pathogenesis of ASD.

## Supplementary Information

**Additional file 1: Figure 1**. Determination of soft-thresholding power in WGCNA. (A) The result of the scale-free fit index for various soft-thresholding powers (β). (B) The result of the mean connectivity for various soft-thresholding powers.

**Additional file 2: Figure 2**. Cluster dendrogram of WGCNA. Dendrogram of 11,617 detected genes in RNA-seq clustered based on a dissimilarity measure (1 – TOM). The modules were merged with setting a cut height value of ‘0.25′, a deep split of ‘2′, and a minimum module size of ‘30′.

**Additional file 3: Figure 3**. The significant module eigengene revealed in WGCNA. The figure represents the correlation between mRNA module eigengenes and phenotypic traits (trait, age, and sex). Each row represents the module eigengene or ME (the correlation matrix of module and sample, labeled by color).

**Additional file 4: Table S1**. All significantly up- and downregulated genes according to the results of RNA sequencing.

**Additional file 5: Table S2**. Results of gene ontology for the significantly upregulated genes.

**Additional file 6: Table S3**. Results of gene ontology for the significantly downregulated genes.

**Additional file 7: Table S4**. All genes included in the MEbrown4 module.

**Additional file 8: Table S5**. Results of gene ontology for all 479 genes included in the MEbrown4 module.

**Additional file 9: Table S6**. Correlation of gene expression with age and sex.

## Data Availability

The data sets used and/or analyzed during the current study are available from the corresponding author on reasonable request.

## Abbreviations

ASD: Autism spectrum disorder
BP: Biological process
CC: Cellular component
cDNA: Complementary DNA
DEG: Differentially expressed gene
GO: Gene ontology
MF: Molecular function
qPCR: Quantitative PCR
RNA-seq: RNA sequencing
TOM: Topological overlap matrix
WGCNA: Weighted gene correlation network analysis

## References

[1] Elsabbagh M, Divan G, Koh YJ, Kim YS, Kauchali S, Marcin C, Montiel-Nava C, Patel V, Paula CS, Wang C (2012). Global prevalence of autism and other pervasive developmental disorders. Autism Res.

[2] Kim YS, Leventhal BL, Koh YJ, Fombonne E, Laska E, Lim EC, Cheon KA, Kim SJ, Kim YK, Lee H, Song DH, Grinker RR (2011). Prevalence of autism spectrum disorders in a total population sample. Am J Psychiatry.

[3] Baio J, Wiggins L, Christensen DL, Maenner MJ, Daniels J, Warren Z, Kurzius-Spencer M, Zahorodny W, Robinson Rosenberg C, White T (2018). Prevalence of autism spectrum disorder among children aged 8 years—autism and developmental disabilities monitoring network, 11 sites, United States, 2014. MMWR Surveill Summ.

[4] Grove J, Ripke S, Als TD, Mattheisen M, Walters RK, Won H, Pallesen J, Agerbo E, Andreassen OA, Anney R (2019). Identification of common genetic risk variants for autism spectrum disorder. Nat Genet.

[5] An JY, Lin K, Zhu L, Werling DM, Dong S, Brand H, Wang HZ, Zhao X, Schwartz GB, Collins RL, Currall BB, Dastmalchi C, Dea J, Duhn C, Gilson MC, Klei L, Liang L, Markenscoff-Papadimitriou E, Pochareddy S, Ahituv N, Buxbaum JD, Coon H, Daly MJ, Kim YS, Marth GT, Neale BM, Quinlan AR, Rubenstein JL, Sestan N, State MW, Willsey AJ, Talkowski ME, Devlin B, Roeder K, Sanders SJ (2018). Genome-wide de novo risk score implicates promoter variation in autism spectrum disorder. Science.

[6] RKCY, Merico D, Bookman M, LH J, Thiruvahindrapuram B, Patel RV, Whitney J, Deflaux N, Bingham J, Wang Z (2017). Whole genome sequencing resource identifies 18 new candidate genes for autism spectrum disorder. Nat Neurosci.

[7] Ruzzo EK, Perez-Cano L, Jung JY, Wang LK, Kashef-Haghighi D, Hartl C, Singh C, Xu J, Hoekstra JN, Leventhal O (2019). Inherited and de novo genetic risk for autism impacts shared networks. Cell.

[8] Krumm N, O'Roak BJ, Karakoc E, Mohajeri K, Nelson B, Vives L, Jacquemont S, Munson J, Bernier R, Eichler EE (2013). Transmission disequilibrium of small CNVs in simplex autism. Am J Hum Genet.

[9] Pinto D, Pagnamenta AT, Klei L, Anney R, Merico D, Regan R, Conroy J, Magalhaes TR, Correia C, Abrahams BS, Almeida J, Bacchelli E, Bader GD, Bailey AJ, Baird G, Battaglia A, Berney T, Bolshakova N, Bölte S, Bolton PF, Bourgeron T, Brennan S, Brian J, Bryson SE, Carson AR, Casallo G, Casey J, Chung BHY, Cochrane L, Corsello C, Crawford EL, Crossett A, Cytrynbaum C, Dawson G, de Jonge M, Delorme R, Drmic I, Duketis E, Duque F, Estes A, Farrar P, Fernandez BA, Folstein SE, Fombonne E, Freitag CM, Gilbert J, Gillberg C, Glessner JT, Goldberg J, Green A, Green J, Guter SJ, Hakonarson H, Heron EA, Hill M, Holt R, Howe JL, Hughes G, Hus V, Igliozzi R, Kim C, Klauck SM, Kolevzon A, Korvatska O, Kustanovich V, Lajonchere CM, Lamb JA, Laskawiec M, Leboyer M, le Couteur A, Leventhal BL, Lionel AC, Liu XQ, Lord C, Lotspeich L, Lund SC, Maestrini E, Mahoney W, Mantoulan C, Marshall CR, McConachie H, McDougle CJ, McGrath J, McMahon WM, Merikangas A, Migita O, Minshew NJ, Mirza GK, Munson J, Nelson SF, Noakes C, Noor A, Nygren G, Oliveira G, Papanikolaou K, Parr JR, Parrini B, Paton T, Pickles A, Pilorge M, Piven J, Ponting CP, Posey DJ, Poustka A, Poustka F, Prasad A, Ragoussis J, Renshaw K, Rickaby J, Roberts W, Roeder K, Roge B, Rutter ML, Bierut LJ, Rice JP, Salt J, Sansom K, Sato D, Segurado R, Sequeira AF, Senman L, Shah N, Sheffield VC, Soorya L, Sousa I, Stein O, Sykes N, Stoppioni V, Strawbridge C, Tancredi R, Tansey K, Thiruvahindrapduram B, Thompson AP, Thomson S, Tryfon A, Tsiantis J, van Engeland H, Vincent JB, Volkmar F, Wallace S, Wang K, Wang Z, Wassink TH, Webber C, Weksberg R, Wing K, Wittemeyer K, Wood S, Wu J, Yaspan BL, Zurawiecki D, Zwaigenbaum L, Buxbaum JD, Cantor RM, Cook EH, Coon H, Cuccaro ML, Devlin B, Ennis S, Gallagher L, Geschwind DH, Gill M, Haines JL, Hallmayer J, Miller J, Monaco AP, Nurnberger Jr JI, Paterson AD, Pericak-Vance MA, Schellenberg GD, Szatmari P, Vicente AM, Vieland VJ, Wijsman EM, Scherer SW, Sutcliffe JS, Betancur C (2010). Functional impact of global rare copy number variation in autism spectrum disorders. Nature.

[10] Poultney CS, Goldberg AP, Drapeau E, Kou Y, Harony-Nicolas H, Kajiwara Y, De Rubeis S, Durand S, Stevens C, Rehnstrom K (2013). Identification of small exonic CNV from whole-exome sequence data and application to autism spectrum disorder. Am J Hum Genet.

[11] Reichenberg A, Gross R, Weiser M, Bresnahan M, Silverman J, Harlap S, Rabinowitz J, Shulman C, Malaspina D, Lubin G, Knobler HY, Davidson M, Susser E (2006). Advancing paternal age and autism. Arch Gen Psychiatry.

[12] Sandin S, Schendel D, Magnusson P, Hultman C, Suren P, Susser E, Gronborg T, Gissler M, Gunnes N, Gross R (2016). Autism risk associated with parental age and with increasing difference in age between the parents. Mol Psychiatry.

[13] Goldmann JM, Wong WS, Pinelli M, Farrah T, Bodian D, Stittrich AB, Glusman G, Vissers LE, Hoischen A, Roach JC (2016). Parent-of-origin-specific signatures of de novo mutations. Nat Genet.

[14] Jonsson H, Sulem P, Kehr B, Kristmundsdottir S, Zink F, Hjartarson E, Hardarson MT, Hjorleifsson KE, Eggertsson HP, Gudjonsson SA (2017). Parental influence on human germline de novo mutations in 1,548 trios from Iceland. Nature.

[15] Kong A, Frigge ML, Masson G, Besenbacher S, Sulem P, Magnusson G, Gudjonsson SA, Sigurdsson A, Jonasdottir A, Jonasdottir A, Wong WSW, Sigurdsson G, Walters GB, Steinberg S, Helgason H, Thorleifsson G, Gudbjartsson DF, Helgason A, Magnusson OT, Thorsteinsdottir U, Stefansson K (2012). Rate of de novo mutations and the importance of father's age to disease risk. Nature.

[16] Rahbari R, Wuster A, Lindsay SJ, Hardwick RJ, Alexandrov LB, Turki SA, Dominiczak A, Morris A, Porteous D, Smith B (2016). Timing, rates and spectra of human germline mutation. Nat Genet.

[17] Iossifov I, Ronemus M, Levy D, Wang Z, Hakker I, Rosenbaum J, Yamrom B, Lee YH, Narzisi G, Leotta A, Kendall J, Grabowska E, Ma B, Marks S, Rodgers L, Stepansky A, Troge J, Andrews P, Bekritsky M, Pradhan K, Ghiban E, Kramer M, Parla J, Demeter R, Fulton LL, Fulton RS, Magrini VJ, Ye K, Darnell JC, Darnell RB, Mardis ER, Wilson RK, Schatz MC, McCombie WR, Wigler M (2012). De novo gene disruptions in children on the autistic spectrum. Neuron.

[18] Sanders SJ, Murtha MT, Gupta AR, Murdoch JD, Raubeson MJ, Willsey AJ, Ercan-Sencicek AG, DiLullo NM, Parikshak NN, Stein JL (2012). De novo mutations revealed by whole-exome sequencing are strongly associated with autism. Nature.

[19] Morgan JT, Chana G, Pardo CA, Achim C, Semendeferi K, Buckwalter J, Courchesne E, Everall IP (2010). Microglial activation and increased microglial density observed in the dorsolateral prefrontal cortex in autism. Biol Psychiatry.

[20] Tetreault NA, Hakeem AY, Jiang S, Williams BA, Allman E, Wold BJ, Allman JM (2012). Microglia in the cerebral cortex in autism. J Autism Dev Disord.

[21] Vargas DL, Nascimbene C, Krishnan C, Zimmerman AW, Pardo CA (2005). Neuroglial activation and neuroinflammation in the brain of patients with autism. Ann Neurol.

[22] Suzuki K, Sugihara G, Ouchi Y, Nakamura K, Futatsubashi M, Takebayashi K, Yoshihara Y, Omata K, Matsumoto K, Tsuchiya KJ, Iwata Y, Tsujii M, Sugiyama T, Mori N (2013). Microglial activation in young adults with autism spectrum disorder. JAMA Psychiatry.

[23] Takano T (2015). Role of microglia in autism: recent advances. Dev Neurosci.

[24] Rodriguez JI, Kern JK (2011). Evidence of microglial activation in autism and its possible role in brain underconnectivity. Neuron Glia Biol.

[25] Ashwood P, Enstrom A, Krakowiak P, Hertz-Picciotto I, Hansen RL, Croen LA, Ozonoff S, Pessah IN, Van de Water J (2008). Decreased transforming growth factor beta1 in autism: a potential link between immune dysregulation and impairment in clinical behavioral outcomes. J Neuroimmunol.

[26] Ashwood P, Krakowiak P, Hertz-Picciotto I, Hansen R, Pessah IN, Van de Water J (2011). Associations of impaired behaviors with elevated plasma chemokines in autism spectrum disorders. J Neuroimmunol.

[27] Croonenberghs J, Bosmans E, Deboutte D, Kenis G, Maes M (2002). Activation of the inflammatory response system in autism. Neuropsychobiology.

[28] Tylee DS, Hess JL, Quinn TP, Barve R, Huang H, Zhang-James Y, Chang J, Stamova BS, Sharp FR, Hertz-Picciotto I, Faraone SV, Kong SW, Glatt SJ (2017). Blood transcriptomic comparison of individuals with and without autism spectrum disorder: A combined-samples mega-analysis. Am J Med Genet B Neuropsychiatr Genet.

[29] Saffari A, Arno M, Nasser E, Ronald A, Wong CCY, Schalkwyk LC, Mill J, Dudbridge F, Meaburn EL (2019). RNA sequencing of identical twins discordant for autism reveals blood-based signatures implicating immune and transcriptional dysregulation. Mol Autism.

[30] Albantakis L, Brandi ML, Zillekens IC, Henco L, Weindel L, Thaler H, Schliephake L, Timmermans B, Schilbach L (2020). Alexithymic and autistic traits: Relevance for comorbid depression and social phobia in adults with and without autism spectrum disorder. Autism.

[31] Rulten SL, Hodder E, Ripley TL, Stephens DN, Mayne LV (2008). Alcohol induces DNA damage and the Fanconi anemia D2 protein implicating FANCD2 in the DNA damage response pathways in brain. Alcohol Clin Exp Res.

[32] Robison AJ, Nestler EJ (2011). Transcriptional and epigenetic mechanisms of addiction. Nat Rev Neurosci.

[33] CL, MR, PD, SR, KG, SB (2012). Autism diagnostic observation schedule–2nd edition (ADOS-2).

[34] Lord C, Rutter M, Le Couteur A (1994). Autism diagnostic interview-revised: a revised version of a diagnostic interview for caregivers of individuals with possible pervasive developmental disorders. J Autism Dev Disord.

[35] Livak KJ, Schmittgen TD (2001). Analysis of relative gene expression data using real-time quantitative PCR and the 2(-Delta Delta C(T)) method. Methods.

[36] Trapnell C, Pachter L, Salzberg SL (2009). TopHat: discovering splice junctions with RNA-Seq. Bioinformatics.

[37] Trapnell C, Williams BA, Pertea G, Mortazavi A, Kwan G, van Baren MJ, Salzberg SL, Wold BJ, Pachter L (2010). Transcript assembly and quantification by RNA-Seq reveals unannotated transcripts and isoform switching during cell differentiation. Nat Biotechnol.

[38] Robinson MD, McCarthy DJ, Smyth GK (2010). edgeR: a Bioconductor package for differential expression analysis of digital gene expression data. Bioinformatics.

[39] Love MI, Huber W, Anders S (2014). Moderated estimation of fold change and dispersion for RNA-seq data with DESeq2. Genome Biol.

[40] Langfelder P, Horvath S (2008). WGCNA: an R package for weighted correlation network analysis. BMC Bioinformatics.

[41] Bindea G, Mlecnik B, Hackl H, Charoentong P, Tosolini M, Kirilovsky A, Fridman WH, Pages F, Trajanoski Z, Galon J (2009). ClueGO: a Cytoscape plug-in to decipher functionally grouped gene ontology and pathway annotation networks. Bioinformatics.

[42] Hernandez-Coro A, Sanchez-Hernandez BE, Montes S, Martinez-Lazcano JC, Gonzalez-Guevara E, Perez-Severiano F (2021). Alterations in gene expression due to chronic lead exposure induce behavioral changes. Neurosci Biobehav Rev.

[43] Jyonouchi H, Geng L, Davidow AL (2014). Cytokine profiles by peripheral blood monocytes are associated with changes in behavioral symptoms following immune insults in a subset of ASD subjects: an inflammatory subtype?. J Neuroinflammation.

[44] Li Q, Han Y, Dy ABC, Hagerman RJ (2017). The gut microbiota and autism spectrum disorders. Front Cell Neurosci.

[45] Jyonouchi H, Sun S, Le H (2001). Proinflammatory and regulatory cytokine production associated with innate and adaptive immune responses in children with autism spectrum disorders and developmental regression. J Neuroimmunol.

[46] Molloy CA, Morrow AL, Meinzen-Derr J, Schleifer K, Dienger K, Manning-Courtney P, Altaye M, Wills-Karp M (2006). Elevated cytokine levels in children with autism spectrum disorder. J Neuroimmunol.

[47] Ashwood P, Krakowiak P, Hertz-Picciotto I, Hansen R, Pessah IN, Van de Water J (2011). Altered T cell responses in children with autism. Brain Behav Immun.

[48] Ashwood P, Anthony A, Torrente F, Wakefield AJ (2004). Spontaneous mucosal lymphocyte cytokine profiles in children with autism and gastrointestinal symptoms: mucosal immune activation and reduced counter regulatory interleukin-10. J Clin Immunol.

[49] Meltzer A, Van de Water J (2017). The role of the immune system in autism spectrum disorder. Neuropsychopharmacology.

[50] Perry VH, Teeling J (2013). Microglia and macrophages of the central nervous system: the contribution of microglia priming and systemic inflammation to chronic neurodegeneration. Semin Immunopathol.

[51] Lintas C, Sacco R, Persico AM (2012). Genome-wide expression studies in autism spectrum disorder, Rett syndrome, and Down syndrome. Neurobiol Dis.

[52] Enstrom AM, Lit L, Onore CE, Gregg JP, Hansen RL, Pessah IN, Hertz-Picciotto I, Van de Water JA, Sharp FR, Ashwood P (2009). Altered gene expression and function of peripheral blood natural killer cells in children with autism. Brain Behav Immun.

[53] Siniscalco D, Mijatovic T, Bosmans E, Cirillo A, Kruzliak P, Lombardi VC, De Meirleir K, Antonucci N (2016). Decreased numbers of CD57+CD3- cells identify potential innate immune differences in patients with autism spectrum disorder. In Vivo.

[54] Whiteley P, Carr K, Shattock P (2019). Is Autism Inborn And Lifelong For Everyone?. Neuropsychiatr Dis Treat.

