# Supplementary figures and images for: Identification of aberrant innate and adaptive immunity based on changes in global gene expression in the blood of adults with autism spectrum disorder
Source: J Neuroinflammation. 2021 Apr 30;18:102. doi: 10.1186/s12974-021-02154-7 (PMC8086363; doi:10.1186/s12974-021-02154-7)

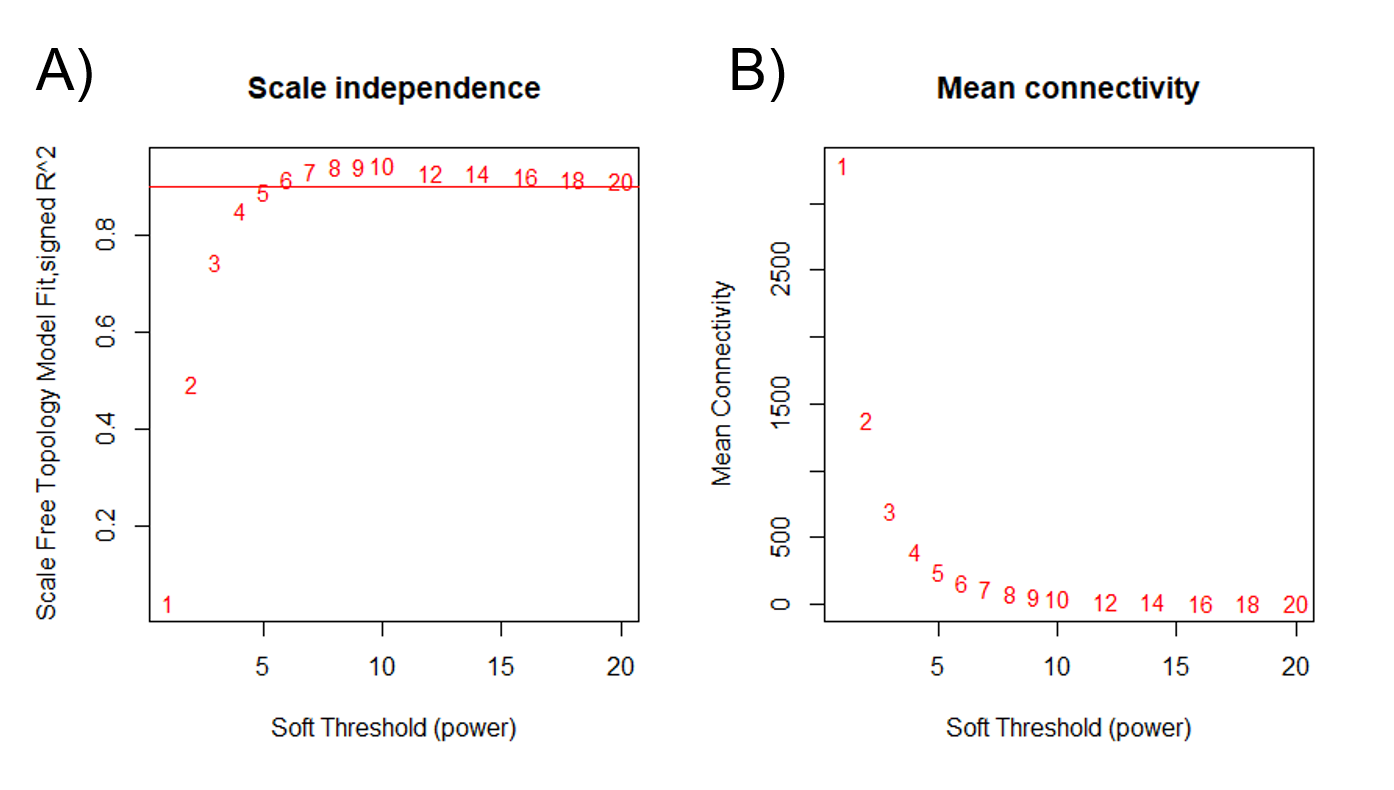

Supplement: Supplementary file 1 — Additional file 1: Figure 1. Determination of soft-thresholding power in WGCNA. (A) The result of the scale-free fit index for various soft-thresholding powers (β). (B) The result of the mean connectivity for various soft-thresholding powers. [file 12974_2021_2154_MOESM1_ESM.tif]

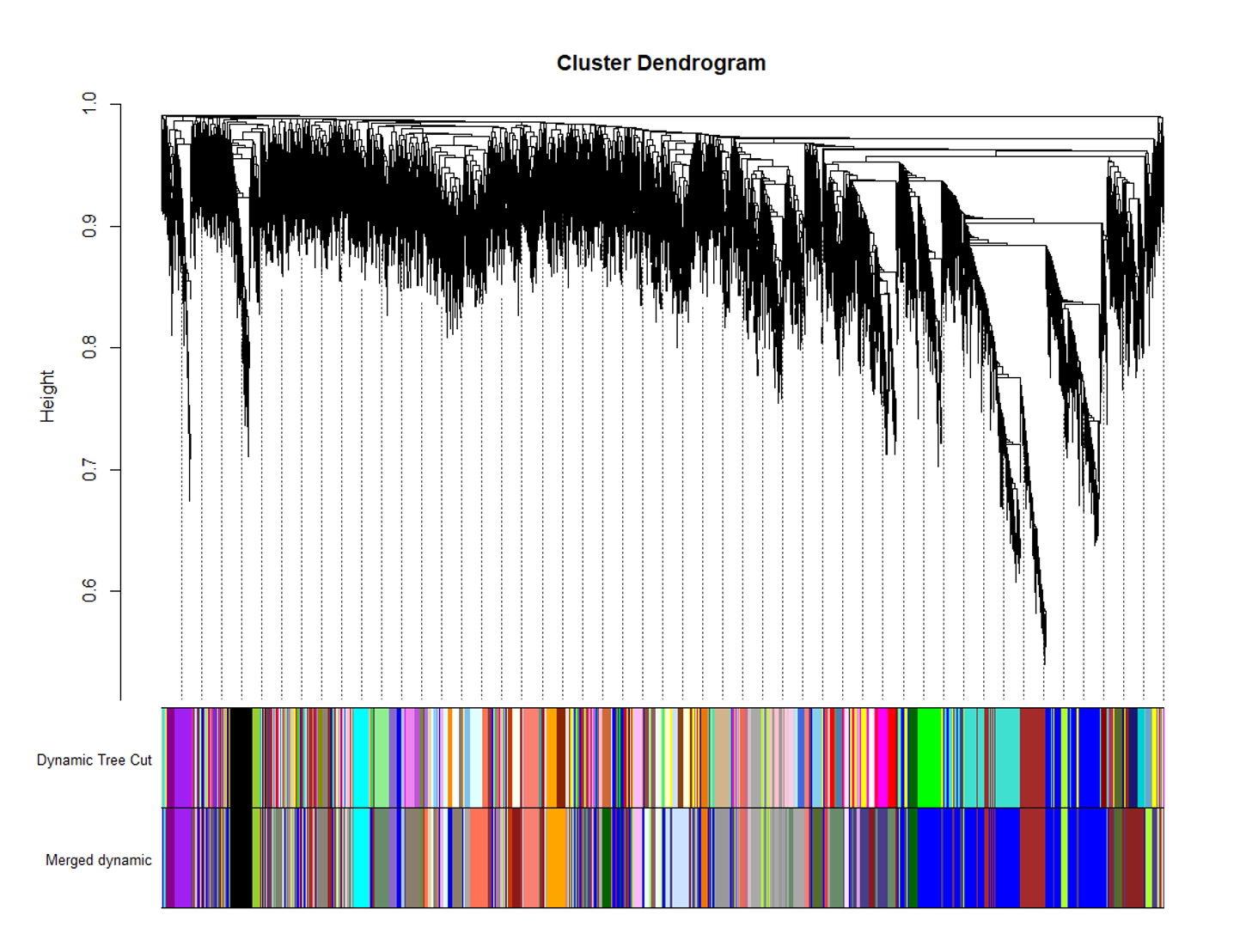

Supplement: Supplementary file 2 — Additional file 2: Figure 2. Cluster dendrogram of WGCNA. Dendrogram of 11,617 detected genes in RNA-seq clustered based on a dissimilarity measure (1 – TOM). The modules were merged with setting a cut height value of ‘0.25′, a deep split of ‘2′, and a minimum module size of ‘30′. [file 12974_2021_2154_MOESM2_ESM.tif]

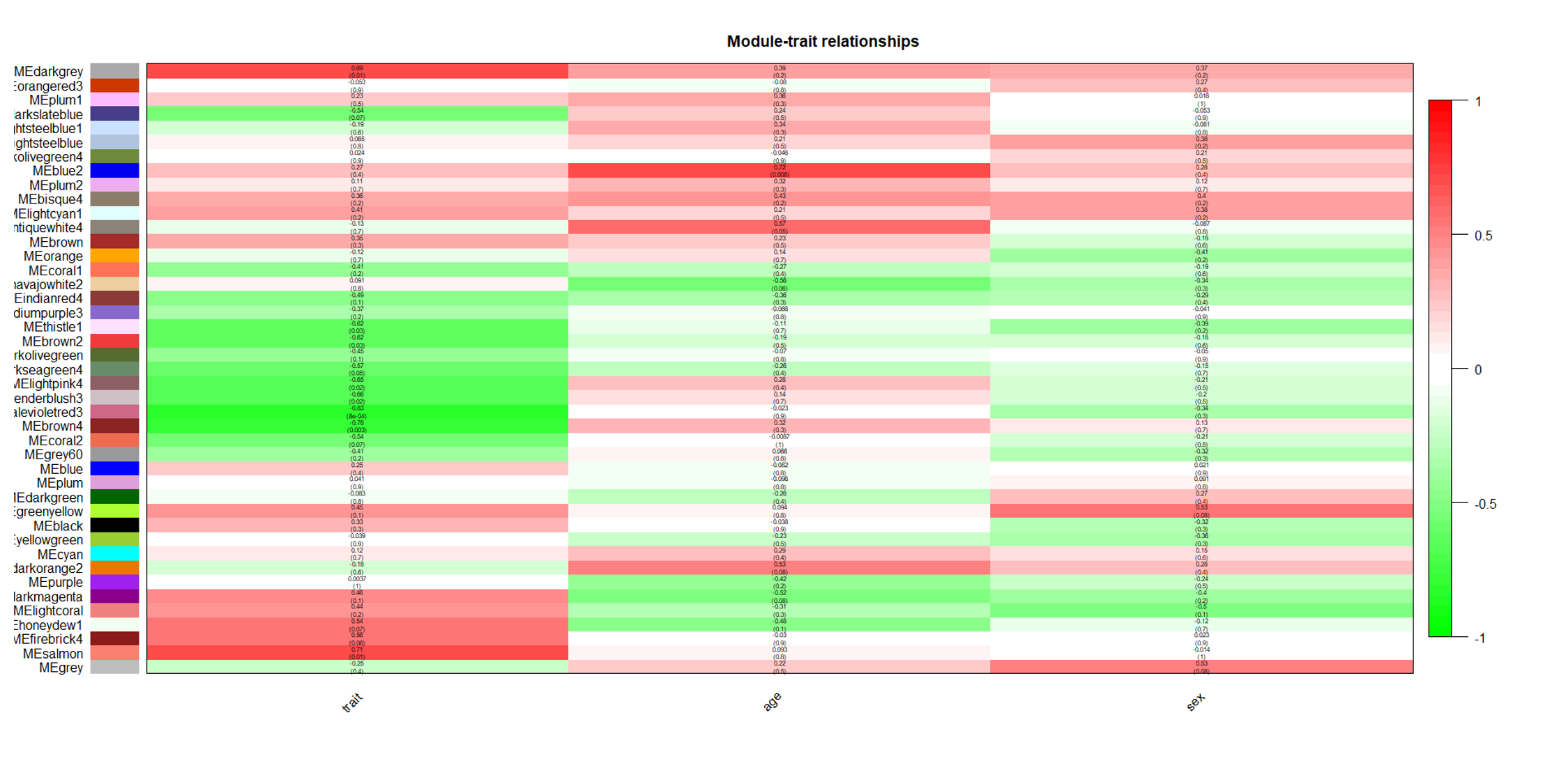

Supplement: Supplementary file 3 — Additional file 3: Figure 3. The significant module eigengene revealed in WGCNA. The figure represents the correlation between mRNA module eigengenes and phenotypic traits (trait, age, and sex). Each row represents the module eigengene or ME (the correlation matrix of module and sample, labeled by color). [file 12974_2021_2154_MOESM3_ESM.tif]
