# Supplementary material for: Identification of aberrant innate and adaptive immunity based on changes in global gene expression in the blood of adults with autism spectrum disorder
Source: J Neuroinflammation. 2021 Apr 30;18:102. doi: 10.1186/s12974-021-02154-7 (PMC8086363; doi:10.1186/s12974-021-02154-7)
Supplement: Supplementary file 4 — Additional file 4: Table S1. All significantly up- and downregulated genes according to the results of RNA sequencing. [file 12974_2021_2154_MOESM4_ESM.docx]

| Gene symbol | Locus | ASD FPKM average value | Ct FPKM average value | Fold change | p value | q value |
| --- | --- | --- | --- | --- | --- | --- |
| **Upregulated genes** | | | | | | |
| *ETV7* | chr6:36354220-36387800 | 12.8652 | 2.18622 | 5.884678 | 5.00E-05 | 0.004845 |
| *BATF2* | chr11:64987944-64997045 | 13.3396 | 2.61341 | 5.104289 | 5.00E-05 | 0.004845 |
| *SERPING1* | chr11:57597553-57614853 | 24.502 | 5.10407 | 4.800483 | 5.00E-05 | 0.004845 |
| *CD274* | chr9:5450502-5470567 | 10.0294 | 2.53744 | 3.952566 | 5.00E-05 | 0.004845 |
| *FCGR1A* | chr1:149782688-149812373 | 16.4805 | 4.31197 | 3.822035 | 0.0005 | 0.024958 |
| *WARS* | chr14:100333787-100376343 | 125.775 | 38.7785 | 3.243421 | 5.00E-05 | 0.004845 |
| *CASP5* | chr11:104994239-105023168 | 18.9458 | 6.30641 | 3.004213 | 5.00E-05 | 0.004845 |
| *PI3* | chr20:45174898-45176544 | 189.799 | 64.3728 | 2.948435 | 5.00E-05 | 0.004845 |
| *FCGR1B* | chr1:121087344-121097161 | 29.3301 | 10.5953 | 2.768218 | 5.00E-05 | 0.004845 |
| *TMEM176B* | chr7:150791287-150805120 | 54.7088 | 20.312 | 2.693423 | 5.00E-05 | 0.004845 |
| *GBP1* | chr1:89052303-89065360 | 56.655 | 21.5723 | 2.626285 | 0.0002 | 0.012551 |
| *KIAA1324* | chr1:109113962-109206781 | 11.3314 | 4.35301 | 2.603118 | 5.00E-05 | 0.004845 |
| *ADGRE1* | chr19:6887548-6940453 | 23.8222 | 9.88847 | 2.409089 | 5.00E-05 | 0.004845 |
| *HLA-DRB5* | chr6:32517376-32530229 | 133.578 | 55.4845 | 2.407483 | 5.00E-05 | 0.004845 |
| *DSC2* | chr18:31065973-31162856 | 11.5088 | 4.97141 | 2.314997 | 5.00E-05 | 0.004845 |
| *MAFB* | chr20:40685847-40689240 | 18.1817 | 7.98773 | 2.276204 | 5.00E-05 | 0.004845 |
| *PLSCR1* | chr3:146515179-146544841 | 38.5497 | 17.1969 | 2.241666 | 5.00E-05 | 0.004845 |
| *ANXA3* | chr4:78551587-78610451 | 16.1477 | 7.42016 | 2.176193 | 0.0001 | 0.007662 |
| *LPCAT2* | chr16:55509000-55586670 | 27.6156 | 12.7193 | 2.171157 | 5.00E-05 | 0.004845 |
| *KCNJ2* | chr17:70169534-70180042 | 43.311 | 20.0467 | 2.160505 | 5.00E-05 | 0.004845 |
| *SORT1* | chr1:109309565-109397941 | 17.9638 | 8.32725 | 2.157231 | 5.00E-05 | 0.004845 |
| *APOL1* | chr22:36253070-36267531 | 25.6837 | 11.9528 | 2.14876 | 5.00E-05 | 0.004845 |
| *SCARF1* | chr17_KI270861v1_alt:63885-75817 | 23.8853 | 11.2045 | 2.13176 | 0.00015 | 0.010189 |
| *VWCE* | chr11:61258285-61295316 | 10.4086 | 4.9563 | 2.100075 | 5.00E-05 | 0.004845 |
| *ODF3B* | chr22:50530408-50532579 | 21.3749 | 10.274 | 2.080485 | 0.00095 | 0.038168 |
| *TYMP* | chr22:50508215-50530085 | 225.98 | 108.881 | 2.075477 | 0.0011 | 0.040719 |
| *TNFAIP6* | chr2:151337825-151380048 | 17.7012 | 8.65096 | 2.046154 | 0.00065 | 0.028938 |
| *DOCK4* | chr7:111726107-112206407 | 9.09748 | 4.45457 | 2.04228 | 0.0001 | 0.007662 |
| *PLBD1* | chr12:14503662-14696585 | 73.6087 | 36.6336 | 2.009322 | 5.00E-05 | 0.004845 |
| *PLD4* | chr14:104924815-104933236 | 13.5779 | 6.76092 | 2.008292 | 0.0003 | 0.016895 |
| *MARCKS* | chr6:113857334-113863475 | 34.7919 | 17.5713 | 1.980041 | 5.00E-05 | 0.004845 |
| *LTA4H* | chr12:96000752-96043520 | 75.204 | 38.1706 | 1.970207 | 5.00E-05 | 0.004845 |
| *GK* | chrX:30653358-30731460 | 32.7705 | 16.782 | 1.952717 | 0.0004 | 0.021255 |
| *UBE2L6* | chr11:57551654-57568330 | 168.17 | 86.1815 | 1.951347 | 0.00075 | 0.031882 |
| *NAPSB* | chr19:50333799-50344748 | 36.3795 | 18.7491 | 1.940333 | 0.00015 | 0.010189 |
| *IFIT3* | chr10:89327818-89340968 | 56.7405 | 29.5738 | 1.918607 | 5.00E-05 | 0.004845 |
| *CRAT* | chr9:129094793-129110791 | 12.0209 | 6.26664 | 1.918237 | 0.00025 | 0.014708 |
| *STK38L* | chr12:27244144-27325957 | 9.91341 | 5.19911 | 1.906751 | 5.00E-05 | 0.004845 |
| *PSTPIP2* | chr18:45983535-46072284 | 19.4139 | 10.2102 | 1.901422 | 5.00E-05 | 0.004845 |
| *NAIP* | chr5_KI270897v1_alt:49475-819690 | 16.3485 | 8.63023 | 1.89433 | 5.00E-05 | 0.004845 |
| *GSN* | chr9:121268101-121332842 | 92.7115 | 49.0118 | 1.891616 | 0.00015 | 0.010189 |
| *ALDH2* | chr12:111766886-111809985 | 40.1123 | 21.2902 | 1.884073 | 5.00E-05 | 0.004845 |
| *PIK3AP1* | chr10:96593311-96720522 | 78.015 | 41.7312 | 1.869465 | 0.0009 | 0.036833 |
| *NAPRT* | chr8_KI270816v1_alt:64824-68391 | 22.4147 | 12.0028 | 1.867456 | 0.0004 | 0.021255 |
| *OSCAR* | chr19_KI270938v1_alt:69045-77107 | 37.1844 | 20.051 | 1.854491 | 5.00E-05 | 0.004845 |
| *ALOX15* | chr17:4630918-4641665 | 12.8903 | 6.97236 | 1.848771 | 0.00075 | 0.031882 |
| *LMNB1* | chr5:126776622-126837020 | 29.059 | 15.9667 | 1.819975 | 0.0001 | 0.007662 |
| *SNX10* | chr7:26291894-26374329 | 28.352 | 15.6189 | 1.815237 | 5.00E-05 | 0.004845 |
| *LINC01410* | chr9:62801464-62813486 | 11.3631 | 6.29363 | 1.805492 | 0.00075 | 0.031882 |
| *HLA-DQB1* | chr6_GL000255v2_alt:3853368-3860969 | 74.6309 | 41.3712 | 1.803934 | 0.00045 | 0.022985 |
| *PRCP* | chr11:82824366-82900515 | 49.4178 | 27.4107 | 1.802865 | 5.00E-05 | 0.004845 |
| *FCRLA* | chr1:161706971-161714352 | 12.2239 | 6.80819 | 1.79547 | 0.0006 | 0.027454 |
| *GRN* | chr17:44345085-44353106 | 197.267 | 110.341 | 1.787794 | 0.00015 | 0.010189 |
| *FFAR2* | chr19:35449714-35451767 | 165.249 | 92.8326 | 1.780075 | 0.0014 | 0.046589 |
| *MARCH1* | chr4:163524297-164383255 | 10.445 | 5.868 | 1.779993 | 5.00E-05 | 0.004845 |
| *RALGPS2* | chr1:178725146-178921842 | 10.5026 | 5.94743 | 1.765906 | 0.0001 | 0.007662 |
| *CYBB* | chrX:37780016-37813461 | 131.542 | 74.6436 | 1.762268 | 0.00055 | 0.026647 |
| *IFI30* | chr19:18173779-18178124 | 617.695 | 350.948 | 1.760076 | 0.0015 | 0.048928 |
| *SEMA4A* | chr1:156149943-156177751 | 51.8373 | 29.4614 | 1.759499 | 5.00E-05 | 0.004845 |
| *SH2B2* | chr7:102285061-102321711 | 18.4163 | 10.5027 | 1.753482 | 0.0002 | 0.012551 |
| *RTN1* | chr14:59595975-59870839 | 10.1121 | 5.77327 | 1.751538 | 0.0004 | 0.021255 |
| *OAS1* | chr12:112906933-112919907 | 38.6196 | 22.121 | 1.745834 | 0.00035 | 0.019218 |
| *HK2* | chr2:74832654-74893354 | 21.5567 | 12.3671 | 1.743068 | 0.00015 | 0.010189 |
| *SEC24D* | chr4:118722822-118836171 | 12.552 | 7.214 | 1.73995 | 0.0001 | 0.007662 |
| *SAMD9L* | chr7:93130053-93148388 | 21.9865 | 12.6594 | 1.736773 | 0.0015 | 0.048928 |
| *RCBTB2* | chr13:48488962-48533256 | 21.2021 | 12.2453 | 1.731448 | 0.0001 | 0.007662 |
| *FIG4* | chr6:109691220-109825431 | 11.8142 | 6.83798 | 1.727732 | 0.00045 | 0.022985 |
| *FGL2* | chr7:77122616-77295204 | 115.599 | 66.918 | 1.727472 | 0.00045 | 0.022985 |
| *VAMP3* | chr1:7771268-7781432 | 31.9738 | 18.521 | 1.726354 | 0.0001 | 0.007662 |
| *CAPZA2* | chr7:116862508-116919259 | 23.21 | 13.4611 | 1.724228 | 0.0002 | 0.012551 |
| *FAM45A* | chr10:119104064-119137984 | 12.2142 | 7.12293 | 1.714772 | 0.0011 | 0.040719 |
| *LILRA6* | chr19_KI270938v1_alt:191260-217825 | 83.3862 | 48.6724 | 1.713213 | 0.0006 | 0.027454 |
| *HSPA1A* | chr6_GL000255v2_alt:3071341-3073770 | 43.4316 | 25.3792 | 1.711307 | 0.0001 | 0.007662 |
| *DUSP6* | chr12:89347824-89352859 | 87.9533 | 51.7349 | 1.700077 | 0.00035 | 0.019218 |
| *KCTD12* | chr13:76880168-76886405 | 32.9997 | 19.4157 | 1.69964 | 0.0001 | 0.007662 |
| *MCTP1* | chr5:94705536-95284575 | 11.2337 | 6.65043 | 1.689169 | 0.00075 | 0.031882 |
| *FAM129B* | chr9:127505337-127579007 | 12.3176 | 7.29835 | 1.687724 | 0.0006 | 0.027454 |
| *ZNF438* | chr10:30844635-31031937 | 11.1102 | 6.5831 | 1.687685 | 0.0006 | 0.027454 |
| *RGS18* | chr1:192158461-192185815 | 20.8225 | 12.3904 | 1.680535 | 0.00075 | 0.031882 |
| *JAK2* | chr9:4985244-5128183 | 22.9263 | 13.6617 | 1.678144 | 0.0002 | 0.012551 |
| *ACSL4* | chrX:109641334-109733392 | 22.1667 | 13.2286 | 1.675665 | 0.00015 | 0.010189 |
| *CYFIP1* | chr15_KI270848v1_alt:743-77414 | 11.6952 | 7.02693 | 1.66434 | 0.00045 | 0.022985 |
| *GNB4* | chr3:179396087-179451583 | 15.5288 | 9.33739 | 1.663077 | 5.00E-05 | 0.004845 |
| *IL1B* | chr2:112829759-112836779 | 45.8437 | 27.592 | 1.661485 | 0.0005 | 0.024958 |
| *TCL1A* | chr14:95709966-95714196 | 40.0849 | 24.1617 | 1.659026 | 0.001 | 0.03922 |
| *C9orf72* | chr9:27546545-27573866 | 28.5535 | 17.2295 | 1.657245 | 0.0006 | 0.027454 |
| *CAPG* | chr2:85394747-85414074 | 74.7775 | 46.2054 | 1.618371 | 0.00035 | 0.019218 |
| *CYP1B1* | chr2:38067602-38076181 | 9.28941 | 5.74726 | 1.61632 | 0.0012 | 0.04274 |
| *HLA-DRB1* | chr6_GL000256v2_alt:3851135-3993866 | 279.418 | 173.636 | 1.609217 | 0.00105 | 0.039991 |
| *PLXNB2* | chr22:50274978-50307572 | 24.5372 | 15.2624 | 1.607689 | 0.00045 | 0.022985 |
| *ZNF467* | chr7:149764363-149773206 | 53.5954 | 33.3873 | 1.605263 | 0.0001 | 0.007662 |
| *RALB* | chr2:120252837-120294710 | 51.8517 | 32.3738 | 1.601656 | 0.0012 | 0.04274 |
| *ANXA5* | chr4:121667996-121696992 | 69.0065 | 43.1218 | 1.600269 | 0.0001 | 0.007662 |
| *SAMD9* | chr7:93099512-93118023 | 10.8918 | 6.80802 | 1.599848 | 0.00105 | 0.039991 |
| *CEBPA* | chr19:33299933-33302564 | 21.4689 | 13.4247 | 1.599209 | 0.00105 | 0.039991 |
| *CD14* | chr5:140631727-140633701 | 208.645 | 130.601 | 1.597576 | 0.0014 | 0.046589 |
| *IFIT2* | chr10:89301948-89309276 | 62.5541 | 39.1919 | 1.596098 | 0.0014 | 0.046589 |
| *CCR2* | chr3:46353743-46360922 | 43.0555 | 27.1263 | 1.587223 | 0.00065 | 0.028938 |
| *HPSE* | chr4:83292460-83335153 | 12.8198 | 8.08003 | 1.586603 | 0.0006 | 0.027454 |
| *IRF5* | chr7:128937936-128950042 | 22.3774 | 14.1075 | 1.586206 | 0.00115 | 0.041864 |
| *FES* | chr15:90884434-90895776 | 52.3121 | 33.014 | 1.584543 | 0.00095 | 0.038168 |
| *LGALS9* | chr17:27631147-27649560 | 94.236 | 59.4763 | 1.584429 | 0.00065 | 0.028938 |
| *DAPP1* | chr4:99816823-99870189 | 37.5498 | 23.776 | 1.579315 | 0.0006 | 0.027454 |
| *RNPEP* | chr1:201982637-202006147 | 27.8821 | 17.6577 | 1.579034 | 0.0014 | 0.046589 |
| *NDST1* | chr5:150497777-150558211 | 15.1364 | 9.59759 | 1.577104 | 0.00115 | 0.041864 |
| *FCER1G* | chr1:161215296-161219248 | 268.218 | 170.232 | 1.575603 | 0.0007 | 0.030955 |
| *TLR1* | chr4:38796254-38804791 | 72.7755 | 46.2488 | 1.573565 | 0.00125 | 0.043349 |
| *TSPO* | chr22:43151513-43163242 | 166.072 | 105.694 | 1.571253 | 0.0006 | 0.027454 |
| *HIATL1* | chr9:94374550-94460920 | 30.5902 | 19.4716 | 1.571016 | 0.0011 | 0.040719 |
| *PLIN3* | chr19:4838333-4867768 | 41.8812 | 26.7422 | 1.566109 | 0.0012 | 0.04274 |
| *NPC2* | chr14:74479939-74493381 | 118.633 | 75.9808 | 1.561355 | 0.001 | 0.03922 |
| *SNX13* | chr7:17790760-17940508 | 9.03223 | 5.79677 | 1.558149 | 0.00125 | 0.043349 |
| *AHR* | chr7:17298651-17346151 | 11.8651 | 7.61763 | 1.557584 | 0.00125 | 0.043349 |
| *ALDH3B1* | chr11:68008546-68029282 | 20.9768 | 13.5218 | 1.551332 | 0.00145 | 0.04801 |
| *UBE2D1* | chr10:58334978-58370753 | 20.5804 | 13.287 | 1.548912 | 0.00155 | 0.049819 |
| *TGFBI* | chr5:136028894-136063818 | 55.7348 | 36.6289 | 1.521607 | 0.0014 | 0.046589 |
| *TMEM30A* | chr6:75252921-75284916 | 26.6222 | 17.5624 | 1.515863 | 0.00155 | 0.049819 |
| **Downregulated genes** | | | | | | |
| *RPSAP58* | chr19:23763013-23828117 | 3.19528 | 10.7714 | 0.296645 | 0.00025 | 0.014708 |
| *GZMK* | chr5:55024278-55034132 | 6.6229 | 20.9275 | 0.316469 | 5.00E-05 | 0.004845 |
| *KLRB1* | chr12:9595273-9607901 | 7.52223 | 22.75 | 0.330647 | 0.00025 | 0.014708 |
| *GZMA* | chr5:55102645-55110252 | 9.13986 | 23.5862 | 0.387509 | 0.00015 | 0.010189 |
| *DUSP2* | chr2:96143168-96145440 | 6.79868 | 16.8911 | 0.402501 | 5.00E-05 | 0.004845 |
| *GNLY* | chr2:85694290-85698851 | 143.126 | 348.618 | 0.410553 | 5.00E-05 | 0.004845 |
| *A2M-AS1* | chr12:9065176-9115962 | 7.10154 | 16.7187 | 0.424766 | 0.00015 | 0.010189 |
| *TOMM7* | chr7:22812632-22822852 | 17.1637 | 39.7824 | 0.43144 | 5.00E-05 | 0.004845 |
| *LINC00612* | chr12:9055588-9065070 | 5.26144 | 12.134 | 0.433611 | 5.00E-05 | 0.004845 |
| *SNORD3A* | chr17:19188015-19188232 | 261.405 | 592.336 | 0.441312 | 5.00E-05 | 0.004845 |
| *RPL27* | chr17:42998428-43002954 | 102.75 | 231.872 | 0.443132 | 5.00E-05 | 0.004845 |
| *SCARNA17,SNHG22* | chr18:49814022-50195081 | 155.186 | 349.705 | 0.443763 | 5.00E-05 | 0.004845 |
| *SH2D2A* | chr1:156806242-156881850 | 13.4704 | 30.1362 | 0.446984 | 5.00E-05 | 0.004845 |
| *AUH* | chr9:91213814-91361969 | 3.9694 | 8.8183 | 0.450132 | 0.0015 | 0.048928 |
| *C6orf48* | chr6_GL000255v2_alt:3090754-3095602 | 30.1026 | 66.6218 | 0.451843 | 5.00E-05 | 0.004845 |
| *S1PR5* | chr19:10512741-10517992 | 12.5973 | 27.307 | 0.461321 | 5.00E-05 | 0.004845 |
| *ZBTB16* | chr11:114059708-114250675 | 9.60781 | 20.7545 | 0.462927 | 5.00E-05 | 0.004845 |
| *DBI* | chr2:119366923-119372551 | 5.64276 | 12.1255 | 0.465363 | 0.0009 | 0.036833 |
| *DDIT4* | chr10:72273918-72276039 | 15.168 | 32.3891 | 0.468306 | 5.00E-05 | 0.004845 |
| *RPS27* | chr1:153990762-153992155 | 150.999 | 318.491 | 0.474108 | 5.00E-05 | 0.004845 |
| *CROCCP2* | chr1:16618255-16630906 | 11.6811 | 24.3021 | 0.480662 | 5.00E-05 | 0.004845 |
| *SMYD3* | chr1:245749339-246507342 | 5.53977 | 11.3257 | 0.489133 | 0.001 | 0.03922 |
| *HIST1H1B* | chr6:27866791-27867581 | 25.2533 | 51.5187 | 0.490177 | 0.0001 | 0.007662 |
| *RPL34* | chr4:108620565-108630483 | 16.6471 | 33.5816 | 0.495721 | 0.0003 | 0.016895 |
| *GZMH* | chr14:24606479-24609720 | 37.4345 | 75.2516 | 0.497458 | 5.00E-05 | 0.004845 |
| *ZFYVE28* | chr4:2269596-2418643 | 5.28256 | 10.4822 | 0.503955 | 0.00025 | 0.014708 |
| *TIGIT* | chr3:114293985-114310288 | 6.41144 | 12.7117 | 0.504373 | 5.00E-05 | 0.004845 |
| *RPS7* | chr2:3575262-3580919 | 45.8503 | 90.8804 | 0.504513 | 5.00E-05 | 0.004845 |
| *COL6A2* | chr21:46098118-46132849 | 4.46626 | 8.75257 | 0.51028 | 0.0002 | 0.012551 |
| *EEF1B2* | chr2:206159593-206162929 | 36.7222 | 71.9061 | 0.510697 | 5.00E-05 | 0.004845 |
| *GZMM* | chr19:544033-549920 | 30.8208 | 59.7666 | 0.515686 | 5.00E-05 | 0.004845 |
| *RPL39* | chrX:119786503-119791659 | 118.931 | 230.367 | 0.516268 | 5.00E-05 | 0.004845 |
| *RPL41* | chr12:56116589-56117832 | 460.529 | 879.927 | 0.523372 | 5.00E-05 | 0.004845 |
| *SYTL2* | chr11:85694220-85811159 | 5.23484 | 9.9493 | 0.526152 | 5.00E-05 | 0.004845 |
| *RPS3A* | chr4:151099572-151104652 | 72.0987 | 136.71 | 0.527384 | 0.0001 | 0.007662 |
| *NKG7* | chr19:51371619-51372706 | 158.724 | 298.489 | 0.531758 | 5.00E-05 | 0.004845 |
| *RPS6* | chr9:19376255-19380237 | 299.414 | 560.313 | 0.534369 | 0.0001 | 0.007662 |
| *SKAP1* | chr17:48133439-48430232 | 23.8827 | 44.595 | 0.535547 | 5.00E-05 | 0.004845 |
| *RPS15A* | chr16:18782954-18790334 | 148.021 | 275.5 | 0.537281 | 5.00E-05 | 0.004845 |
| *RDH13* | chr19_KI270938v1_alt:1026805-1052028 | 5.82578 | 10.8122 | 0.538815 | 0.0013 | 0.044847 |
| *MYBL1* | chr8:66562174-66613249 | 4.95518 | 9.17852 | 0.539867 | 5.00E-05 | 0.004845 |
| *RPL26* | chr17:8377515-8383247 | 55.6373 | 102.968 | 0.540336 | 0.0001 | 0.007662 |
| *PDZD4* | chrX:153802165-153830567 | 5.79425 | 10.718 | 0.540609 | 5.00E-05 | 0.004845 |
| *KLRK1* | chr12:10363768-10410146 | 23.4039 | 42.9065 | 0.545463 | 0.0005 | 0.024958 |
| *MIAT* | chr22:26657481-26780893 | 17.7038 | 32.3829 | 0.546702 | 0.0002 | 0.012551 |
| *PYHIN1* | chr1:158931546-158977059 | 11.6816 | 21.362 | 0.54684 | 5.00E-05 | 0.004845 |
| *FGFBP2* | chr4:15960239-15963236 | 29.1481 | 53.195 | 0.547948 | 0.0001 | 0.007662 |
| *GZMB* | chr14:24630955-24634226 | 44.3983 | 80.5946 | 0.550884 | 0.0002 | 0.012551 |
| *TSEN54* | chr17:75516527-75524739 | 10.4692 | 18.9744 | 0.551754 | 0.00055 | 0.026647 |
| *LIG1* | chr19:48115444-48170603 | 6.13955 | 10.9703 | 0.559652 | 0.00015 | 0.010189 |
| *RPL9* | chr4:39454124-39458948 | 92.8792 | 165.176 | 0.562304 | 0.00015 | 0.010189 |
| *CD8B* | chr2:86815336-86895042 | 19.5301 | 34.6123 | 0.564253 | 0.00025 | 0.014708 |
| *ADGRG1* | chr16:57619692-57665039 | 17.2141 | 30.4314 | 0.565669 | 0.0001 | 0.007662 |
| *ADGRG5* | chr16:57542420-57577188 | 9.36261 | 16.5479 | 0.565788 | 5.00E-05 | 0.004845 |
| *RPS18* | chr6_GL000256v2_alt:4720755-4721408 | 449.094 | 791.395 | 0.567471 | 0.0003 | 0.016895 |
| *C9orf78* | chr9:129827284-129835293 | 52.5941 | 92.076 | 0.571203 | 5.00E-05 | 0.004845 |
| *MATK* | chr19:3777968-3801812 | 17.7317 | 30.9204 | 0.573463 | 0.00025 | 0.014708 |
| *FCRL6* | chr1:159800510-159816257 | 12.2355 | 21.1421 | 0.578727 | 0.00055 | 0.026647 |
| *IGSF8* | chr1:160091338-160098828 | 9.17513 | 15.6918 | 0.584709 | 0.001 | 0.03922 |
| *CD3D* | chr11:118339073-118342744 | 30.0986 | 51.464 | 0.584848 | 0.00095 | 0.038168 |
| *CCDC57* | chr17:82101469-82212813 | 6.28483 | 10.7183 | 0.586364 | 0.00135 | 0.046329 |
| *TGFBR3* | chr1:91680342-91906002 | 7.50252 | 12.7512 | 0.588378 | 0.0001 | 0.007662 |
| *KLRD1* | chr12:10304450-10317251 | 14.5127 | 24.5951 | 0.590065 | 0.00015 | 0.010189 |
| *TARP* | chr7:38259642-38273647 | 29.4295 | 49.7617 | 0.591409 | 0.0009 | 0.036833 |
| *CCL5* | chr17_KI270857v1_alt:106023-114905 | 171.33 | 289.016 | 0.592805 | 0.0004 | 0.021255 |
| *SLAMF7* | chr1:160739056-160754818 | 11.8355 | 19.9339 | 0.593737 | 0.0002 | 0.012551 |
| *SAMD3* | chr6:130144301-130365425 | 12.7033 | 21.389 | 0.593917 | 0.00085 | 0.035447 |
| *HIST1H4E* | chr6:26204644-26205021 | 234.375 | 389.82 | 0.601239 | 0.00025 | 0.014708 |
| *DIP2A* | chr21:46458948-46570013 | 20.7316 | 34.0364 | 0.609101 | 0.0014 | 0.046589 |
| *ST6GALNAC6* | chr9:127885320-127905348 | 18.402 | 29.8381 | 0.616728 | 0.0003 | 0.016895 |
| *RPS27A* | chr2:55172550-55235853 | 110.992 | 179.958 | 0.616766 | 0.00075 | 0.031882 |
| *RPL12* | chr9:127447673-127451432 | 334.45 | 536.268 | 0.623662 | 0.00105 | 0.039991 |
| *SLAMF6* | chr1:160485029-160523262 | 16.47 | 26.3434 | 0.625204 | 0.00055 | 0.026647 |
| *PRKCH* | chr14:61321442-61550980 | 45.7168 | 73.0979 | 0.625419 | 0.0008 | 0.03379 |
| *CD2* | chr1:116754463-116769229 | 33.2047 | 52.761 | 0.629342 | 0.00115 | 0.041864 |
| *PRKXP1* | chr15:100547751-100559283 | 5.76208 | 9.13675 | 0.630649 | 0.0003 | 0.016895 |
| *GIMAP7* | chr7:150514856-150521073 | 43.0346 | 68.1205 | 0.631742 | 0.00085 | 0.035447 |
| *CD247* | chr1:167430639-167518610 | 71.9665 | 113.134 | 0.636117 | 0.00125 | 0.043349 |
| *HIST1H1D* | chr6:26234211-26234988 | 103.048 | 161.519 | 0.637993 | 0.0012 | 0.04274 |
| *NLRC3* | chr16:3500944-3577404 | 20.1205 | 31.4734 | 0.639286 | 0.0011 | 0.040719 |
| *ZAP70* | chr2:97713567-97739860 | 55.7111 | 87.0888 | 0.639705 | 0.0011 | 0.040719 |
| *ZNF831* | chr20:59191019-59259112 | 10.794 | 16.7245 | 0.6454 | 0.00105 | 0.039991 |
| *DNAH1* | chr3:52316318-52400497 | 10.0534 | 15.329 | 0.655842 | 0.00125 | 0.043349 |
