# Supplementary material for: Identification of aberrant innate and adaptive immunity based on changes in global gene expression in the blood of adults with autism spectrum disorder
Source: J Neuroinflammation. 2021 Apr 30;18:102. doi: 10.1186/s12974-021-02154-7 (PMC8086363; doi:10.1186/s12974-021-02154-7)
Supplement: Supplementary file 5 — Additional file 5: Table S2. Results of gene ontology for the significantly upregulated genes. [file 12974_2021_2154_MOESM5_ESM.docx]

| GOID | GO Term | Benjamini-Hochberg  P value | Number of genes |
| --- | --- | --- | --- |
| **Biological process** | | | |
| GO:0036151 | phosphatidylcholine acyl-chain remodeling | 0.001226 | 3 |
| GO:0044088 | regulation of vacuole organization | 0.000404 | 4 |
| GO:0046164 | alcohol catabolic process | 0.001090 | 4 |
| GO:0050818 | regulation of coagulation | 0.004210 | 4 |
| GO:0051016 | barbed-end actin filament capping | 0.000913 | 3 |
| GO:0016125 | sterol metabolic process | 0.001612 | 6 |
| GO:1902652 | secondary alcohol metabolic process | 0.001626 | 6 |
| GO:0009595 | detection of biotic stimulus | 0.000349 | 4 |
| GO:0016045 | detection of bacterium | 0.000343 | 3 |
| GO:0009615 | response to virus | 0.000052 | 11 |
| GO:0051607 | defense response to virus | 0.000545 | 8 |
| GO:0060337 | type I interferon signaling pathway | 0.003971 | 4 |
| GO:0002274 | myeloid leukocyte activation | 3.3892E-13 | 28 |
| GO:0002366 | leukocyte activation involved in immune response | 5.4866E-13 | 24 |
| GO:0002444 | myeloid leukocyte mediated immunity | 9.873E-11 | 23 |
| GO:0036230 | granulocyte activation | 6.066E-10 | 21 |
| GO:0043299 | leukocyte degranulation | 7.505E-11 | 23 |
| GO:0045055 | regulated exocytosis | 7.5942E-11 | 27 |
| GO:0043312 | neutrophil degranulation | 1.6179E-09 | 20 |
| GO:0048002 | antigen processing and presentation of peptide antigen | 4.677E-08 | 12 |
| GO:0002474 | antigen processing and presentation of peptide antigen via MHC class I | 0.000016 | 7 |
| GO:0002495 | antigen processing and presentation of peptide antigen via MHC class II | 2.4380E-06 | 8 |
| GO:0006911 | phagocytosis, engulfment | 0.002811 | 5 |
| GO:0002479 | antigen processing and presentation of exogenous peptide antigen via MHC class I, TAP-dependent | 0.002145 | 4 |
| GO:0071346 | cellular response to interferon-gamma | 2.6299E-09 | 13 |
| GO:0060333 | interferon-gamma-mediated signaling pathway | 4.443E-09 | 10 |
| GO:0050777 | negative regulation of immune response | 0.000174 | 7 |
| GO:0002886 | regulation of myeloid leukocyte mediated immunity | 0.001048 | 4 |
| GO:0032649 | regulation of interferon-gamma production | 0.000173 | 6 |
| GO:0032729 | positive regulation of interferon-gamma production | 0.001624 | 4 |
| GO:0043300 | regulation of leukocyte degranulation | 0.000453 | 4 |
| GO:0050868 | negative regulation of T cell activation | 0.007870 | 4 |
| GO:1902105 | regulation of leukocyte differentiation | 0.000282 | 9 |
| GO:0002573 | myeloid leukocyte differentiation | 0.000048 | 9 |
| GO:1902106 | negative regulation of leukocyte differentiation | 0.004991 | 4 |
| GO:0002761 | regulation of myeloid leukocyte differentiation | 0.008567 | 4 |
| GO:0030316 | osteoclast differentiation | 0.001027 | 5 |
| GO:1903409 | reactive oxygen species biosynthetic process | 0.001702 | 5 |
| GO:0002832 | negative regulation of response to biotic stimulus | 0.003971 | 4 |
| GO:0050777 | negative regulation of immune response | 0.000174 | 7 |
| GO:1901570 | fatty acid derivative biosynthetic process | 0.006577 | 4 |
| GO:0002819 | regulation of adaptive immune response | 0.001631 | 6 |
| GO:0032613 | interleukin-10 production | 0.001132 | 4 |
| GO:0032637 | interleukin-8 production | 0.000080 | 6 |
| GO:0042116 | macrophage activation | 0.004730 | 4 |
| GO:0045576 | mast cell activation | 0.001420 | 4 |
| GO:0061900 | glial cell activation | 0.006789 | 3 |
| GO:0071706 | tumor necrosis factor superfamily cytokine production | 0.000009 | 9 |
| GO:1903900 | regulation of viral life cycle | 0.004153 | 5 |
| GO:1903901 | negative regulation of viral life cycle | 0.017735 | 3 |
| GO:0042089 | cytokine biosynthetic process | 0.001702 | 5 |
| GO:0006690 | icosanoid metabolic process | 0.010183 | 4 |
| GO:0033003 | regulation of mast cell activation | 0.000428 | 4 |
| GO:0050727 | regulation of inflammatory response | 0.000005 | 13 |
| GO:0002718 | regulation of cytokine production involved in immune response | 0.004387 | 4 |
| GO:0002886 | regulation of myeloid leukocyte mediated immunity | 0.001048 | 4 |
| GO:0010574 | regulation of vascular endothelial growth factor production | 0.000179 | 4 |
| GO:0032649 | regulation of interferon-gamma production | 0.000173 | 6 |
| GO:0032653 | regulation of interleukin-10 production | 0.001033 | 4 |
| GO:0045428 | regulation of nitric oxide biosynthetic process | 0.007918 | 3 |
| GO:0046596 | regulation of viral entry into host cell | 0.002164 | 3 |
| GO:0046631 | alpha-beta T cell activation | 0.003496 | 5 |
| GO:0050729 | positive regulation of inflammatory response | 0.000174 | 7 |
| GO:1903555 | regulation of tumor necrosis factor superfamily cytokine production | 0.000007 | 9 |
| GO:0002824 | positive regulation of adaptive immune response based on somatic recombination of immune receptors built from immunoglobulin superfamily domains | 0.001485 | 5 |
| GO:0032689 | negative regulation of interferon-gamma production | 0.001750 | 3 |
| GO:0032722 | positive regulation of chemokine production | 0.009761 | 3 |
| GO:0032729 | positive regulation of interferon-gamma production | 0.001624 | 4 |
| GO:0032757 | positive regulation of interleukin-8 production | 0.000009 | 6 |
| GO:0042108 | positive regulation of cytokine biosynthetic process | 0.000342 | 5 |
| GO:1903556 | negative regulation of tumor necrosis factor superfamily cytokine production | 0.008758 | 3 |
| GO:0002675 | positive regulation of acute inflammatory response | 0.001694 | 3 |
| GO:0035710 | CD4-positive, alpha-beta T cell activation | 0.022665 | 3 |
| GO:0043300 | regulation of leukocyte degranulation | 0.000453 | 4 |
| GO:0043303 | mast cell degranulation | 0.004522 | 3 |
| GO:0046633 | alpha-beta T cell proliferation | 0.001872 | 3 |
| GO:0050707 | regulation of cytokine secretion | 0.000337 | 8 |
| GO:0050868 | negative regulation of T cell activation | 0.007870 | 4 |
| GO:1902105 | regulation of leukocyte differentiation | 0.000282 | 9 |
| GO:0002827 | positive regulation of T-helper 1 type immune response | 0.000847 | 3 |
| GO:0046634 | regulation of alpha-beta T cell activation | 0.004387 | 4 |
| GO:0050715 | positive regulation of cytokine secretion | 0.001236 | 6 |
| GO:0072606 | interleukin-8 secretion | 0.000179 | 4 |
| GO:1904037 | positive regulation of epithelial cell apoptotic process | 0.001750 | 3 |
| GO:0045765 | regulation of angiogenesis | 0.000161 | 10 |
| GO:0045766 | positive regulation of angiogenesis | 0.001702 | 6 |
| GO:2000484 | positive regulation of interleukin-8 secretion | 0.000016 | 4 |
| GO:0002292 | T cell differentiation involved in immune response | 0.001604 | 4 |
| GO:0031103 | axon regeneration | 0.000892 | 4 |
| GO:2001269 | positive regulation of cysteine-type endopeptidase activity involved in apoptotic signaling pathway | 0.000181 | 3 |
| **Cellular components** | | | |
| GO:1904813 | ficolin-1-rich granule lumen | 0.0004468 | 6 |
| GO:0042581 | specific granule | 0.00034213 | 7 |
| GO:0070820 | tertiary granule | 0.0003406 | 7 |
| GO:1904724 | tertiary granule lumen | 0.0007851 | 4 |
| GO:0035579 | specific granule membrane | 0.02177 | 3 |
| GO:0035580 | specific granule lumen | 0.009264 | 3 |
| GO:0005802 | trans-Golgi network | 0.00013989 | 9 |
| GO:0030139 | endocytic vesicle | 7.8636E-06 | 12 |
| GO:0042613 | MHC class II protein complex | 0.0003678 | 3 |
| GO:0030658 | transport vesicle membrane | 0.0007700 | 7 |
| GO:0030662 | coated vesicle membrane | 0.0004632 | 7 |
| GO:0030666 | endocytic vesicle membrane | 2.2155E-06 | 10 |
| GO:0030136 | clathrin-coated vesicle | 0.0005277 | 7 |
| GO:0032588 | trans-Golgi network membrane | 0.0001852 | 6 |
| GO:0045335 | phagocytic vesicle | 0.0004860 | 6 |
| GO:0071556 | integral component of lumenal side of endoplasmic reticulum membrane | 0.001171 | 3 |
| GO:0012507 | ER to Golgi transport vesicle membrane | 0.0009784 | 4 |
| GO:0030670 | phagocytic vesicle membrane | 0.001740 | 4 |
| **Molecular function** | | | |
| GO:0016803 | ether hydrolase activity | 0.0001216 | 3 |
| GO:0019864 | IgG binding | 0.00008072 | 3 |
| GO:1901981 | phosphatidylinositol phosphate binding | 0.001089 | 6 |
| GO:2001269 | positive regulation of cysteine-type endopeptidase activity involved in apoptotic signaling pathway | 0.00008072 | 3 |
