# Supplementary material for: Identification of aberrant innate and adaptive immunity based on changes in global gene expression in the blood of adults with autism spectrum disorder
Source: J Neuroinflammation. 2021 Apr 30;18:102. doi: 10.1186/s12974-021-02154-7 (PMC8086363; doi:10.1186/s12974-021-02154-7)
Supplement: Supplementary file 6 — Additional file 6: Table S3. Results of gene ontology for the significantly downregulated genes. [file 12974_2021_2154_MOESM6_ESM.docx]

| GOID | GO Term | Benjamini-Hochberg  P value | Number of genes |
| --- | --- | --- | --- |
| **Biological process** | | | |
| GO:0043368 | positive T cell selection | 0.00001435 | 4 |
| GO:0001909 | leukocyte mediated cytotoxicity | 0.00007742 | 5 |
| GO:0002228 | natural killer cell mediated immunity | 0.000006655 | 5 |
| GO:0019083 | viral transcription | 1.0339E-15 | 15 |
| GO:0090150 | establishment of protein localization to membrane | 9.5750E-12 | 15 |
| GO:0006605 | protein targeting | 2.5934E-10 | 15 |
| GO:0000184 | nuclear-transcribed mRNA catabolic process, nonsense-mediated decay | 1.8570E-16 | 14 |
| GO:0006614 | SRP-dependent cotranslational protein targeting to membrane | 1.9217E-17 | 14 |
| **Cellular components** | | | |
| GO:0022625 | cytosolic large ribosomal subunit | 4.8157E-09 | 7 |
| GO:0022627 | cytosolic small ribosomal subunit | 1.7992E-09 | 7 |
| GO:0042101 | T cell receptor complex | 0.00001596 | 6 |
| GO:0042105 | alpha-beta T cell receptor complex | 3.3485E-06 | 3 |
| GO:0022626 | cytosolic ribosome | 5.7048E-17 | 14 |
| GO:0044391 | ribosomal subunit | 4.6884E-14 | 14 |
| **Molecular function** | | | |
| none | | | |
