# Supplementary material for: Identification of aberrant innate and adaptive immunity based on changes in global gene expression in the blood of adults with autism spectrum disorder
Source: J Neuroinflammation. 2021 Apr 30;18:102. doi: 10.1186/s12974-021-02154-7 (PMC8086363; doi:10.1186/s12974-021-02154-7)
Supplement: Supplementary file 7 — Additional file 7: Table S4. All genes included in the MEbrown4 module. [file 12974_2021_2154_MOESM7_ESM.docx]

| *PAPSS2* | *CERS6-AS1* | *ZNF558* | *MDC1* | *STX12* | *ZFYVE28* | *NKIRAS2* | *SLAMF7* |
| --- | --- | --- | --- | --- | --- | --- | --- |
| *SPATA6* | *LOC101928855* | *SNPH* | *BAIAP2-AS1* | *LOC100130872,SPON2* | *IFT27* | *HSD17B4* | *DGKQ* |
| *GAREML* | *OTUD7B* | *TRIM66* | *TARBP2* | *TSHZ1* | *MRPL4* | *KLHDC2* | *HEXB* |
| *PPP1R26* | *SLC16A4* | *UEVLD* | *EAF2* | *NSMCE2* | *SLC25A42* | *KIAA1191* | *VPS35* |
| *TMEM144* | *UBE2E2* | *LGR6* | *CRAT* | *ASL* | *PDZD4* | *TSR2* | *FCRL6* |
| *PMEPA1* | *DEGS2* | *GPATCH2* | *SLC35D1* | *IL21R* | *GMPPA* | *ERCC3* | *LSM7* |
| *FOXO3B* | *NOMO1* | *RGS9* | *DTHD1* | *HCFC1R1* | *RBM27* | *ULK3* | *TRIB2* |
| *MROH6* | *MAGEH1* | *ARFIP2* | *NMNAT1* | *MRM1* | *TANGO6* | *SENP5* | *BTK* |
| *CENPK* | *MARVELD1* | *TRIM3* | *THOC3* | *FUCA1* | *KLHL12* | *KLF16* | *PYHIN1* |
| *FBXL19-AS1* | *PEX12* | *GAB1* | *ENOPH1* | *DPH1* | *MS4A7* | *GNPTAB* | *SAMD3* |
| *LOC101926889* | *MANEA-AS1* | *DPAGT1* | *ITPRIPL1* | *ERMP1* | *PDCD7* | *TRMT1* | *CBLB* |
| *C12orf4* | *C22orf46* | *AK3* | *NMT2* | *MEF2C* | *STAB1* | *ARPC5L* | *SLC3A2* |
| *FZD1* | *SLC38A6* | *AGAP1* | *POLE* | *RMDN3* | *NUP160* | *TBCD* | *DGUOK* |
| *CFL2* | *LOC100506302* | *IRAK2* | *SLC25A26* | *ANKIB1* | *TXNL4A* | *ETV3* | *ABCA2* |
| *CCNB1* | *CDC42BPB* | *ZNF383* | *CAD* | *ZNF264* | *S100PBP* | *CAPN15* | *GALT* |
| *COL9A3* | *FBF1* | *EIF3J-AS1* | *ANAPC1* | *COL6A2* | *LOC100996255* | *SNRPA* | *CHST12* |
| *NTN3* | *EPHA4* | *PRSS23* | *ZBED2* | *ATP10A* | *LSS* | *BMP2K* | *TSG101* |
| *EXD2* | *CACNA2D2* | *MED7* | *FASLG* | *SLC22A18* | *ZNF317* | *ADGRG5* | *CMTM7* |
| *LRRC37A8P* | *PPP1R13B* | *CCDC107* | *GALNT7* | *GATA3* | *RNF135* | *SKIV2L* | *GZMA* |
| *ABCA3* | *SRGAP2B* | *ETFDH* | *TFDP2* | *LDOC1L* | *RRP8* | *GAB3* | *RALGDS* |
| *RNF39* | *RNF165* | *DCP1B* | *CYFIP1* | *MAP3K4* | *TRG-AS1* | *GCN1* | *STAG3L5P-PVRIG2P-PILRB* |
| *EFHC1* | *RIN2* | *LINC00893* | *MOSPD3* | *ECHDC2* | *LAX1* | *ZNF831* | *ESYT2* |
| *SLC25A53* | *AAED1* | *SEPT7P2* | *GDPD5* | *MYBL1* | *DIABLO* | *PCNXL3* | *KLRD1* |
| *PER3* | *CEP89* | *PAK4* | *SNRPB2* | *ACSS2* | *SNAPC5* | *ZNF524* | *DOCK10* |
| *PLEKHA8P1* | *ZNF827* | *GGACT* | *EDRF1* | *SBK1* | *RTCB* | *KANSL3* | *C9orf69* |
| *E2F1* | *ZNF354B* | *FAM133B* | *MARK4* | *SEPN1* | *NRF1* | *ZBTB25* | *SPINT2* |
| *DRAXIN* | *MRPL47* | *TERF1* | *GOLGA1* | *NSUN3* | *PWWP2B* | *METTL9* | *SYNE1* |
| *LOC727896* | *LIMA1* | *LINC01278* | *DPH2* | *AGAP2-AS1* | *MAF* | *MARCKS* | *DAXX* |
| *PDCL3* | *ZNF254* | *BCKDHB* | *GABPB2* | *ASB1* | *NAPRT* | *SLC2A4RG* | *LY9* |
| *PHLDB2* | *MOB4* | *ANKRD13C* | *DENR* | *VPS36* | *TBC1D25* | *NELFCD* | *APOBEC3G* |
| *YES1* | *CRIM1* | *DOPEY1* | *CMKLR1* | *TNPO2* | *ENO2* | *ARIH2* | *BCL11B* |
| *PI16* | *C19orf68* | *DCAF4* | *OSBPL7* | *DSE* | *GTF2B* | *BTBD6* | *CCR2* |
| *PALLD* | *AP4B1-AS1* | *GPRASP1* | *CCDC28B* | *MZB1* | *TXNRD2* | *YWHAG* | *HIST1H2BG* |
| *TCEA3* | *PTCH1* | *HEG1* | *NSMCE4A* | *YPEL2* | *ARHGAP35* | *TTC38* | *BCL9L* |
| *TMEM192* | *DDX20* | *PEX1* | *RNMT* | *NPRL2* | *POU2AF1* | *ATG4B* | *S1PR5* |
| *LGALS9C* | *HOXB4* | *UBOX5* | *HSPBP1* | *FAF2* | *RLTPR* | *ZFAND5* | *C9orf142* |
| *MAP3K13* | *SLC9A7* | *ARHGEF10L* | *HSD17B12* | *C9orf114* | *UTP6* | *NCR1* | *TBX21* |
| *ZDHHC14* | *TMEM116* | *LOC100507639* | *LOC100652999* | *SEC31B* | *C2CD3* | *NARS* | *PLCG1* |
| *FBLN7* | *GPR75* | *PPM1L* | *MYO19* | *NAA40* | *TGFBR3* | *TSEN54* | *PCED1B-AS1* |
| *RABL2A* | *TOX* | *ZNF70* | *MED18* | *TRAF2* | *OSBPL5* | *LONP2* | *TRPV2* |
| *XXYLT1* | *ORC3* | *GK5* | *ADAMTS10* | *SYTL2* | *GARS* | *KLRC4* | *BIRC6* |
| *C1orf216* | *RFC5* | *SIRT5* | *SERPINB8* | *LCMT1* | *STK39* | *LOC103091866* | *ADGRG1* |
| *HLTF* | *PAXIP1* | *FAM105A* | *VSTM1* | *LY6G5B* | *CLEC4A* | *RCOR3* | *PCED1B* |
| *ZNF568* | *FAM179B* | *LOC283788* | *BIN3-IT1* | *MDN1* | *CC2D1B* | *NFATC2* | *MIEN1* |
| *SOGA1* | *ANO8* | *NSUN5P2* | *MRS2* | *EOMES* | *LZTR1* | *KCTD12* | *MATK* |
| *GYLTL1B* | *SLC35A1* | *LOC101928100* | *TMEM245* | *BZRAP1* | *NPC1* | *GBGT1* | *NLRC3* |
| *LOC101927543* | *PTDSS2* | *JAKMIP1* | *NSG1* | *WHSC1* | *ITPR3* | *ATP2B1* | *ST13* |
| *EVI5* | *SYNJ2* | *AARS2* | *LETM1* | *LINC00476* | *TAF1C* | *RAB29* | *ATP2B4* |
| *USP32P2* | *RAB42* | *TNFRSF8* | *POU5F1P4* | *OGFOD2* | *CEBPA* | *PLEKHF1* | *ID2* |
| *GPR55* | *NBPF3* | *ZNF490* | *NTPCR* | *HOPX* | *ALDH3B1* | *KMT2A* | *RBM6* |
| *MIAT* | *IKZF3* | *DPP7* | *SIGIRR* | *INPP4A* | *GZMM* | *LCK* | *CST3* |
| *ATP6AP1* | *SPTAN1* | *PPP3R1* | *WDR83OS* | *EIF2S3* | *GIMAP6* | *ETS1* | *CRIP1* |
| *GSK3A* | *TGFBI* | *ITK* | *SYTL1* | *CD2* | *LAMTOR1* | *HIST1H2BD* | *NCOA4* |
| *MAP3K1* | *HVCN1* | *KLRK1* | *NDUFA3* | *ARL4C* | *TWF2* | *CD247* | *CCL5* |
| *ZDHHC7* | *TNFRSF25* | *MACF1* | *IL2RB* | *ASAH1* | *PRKCH* | *SLC9A3R1* | *NKG7* |
| *SLFN5* | *NISCH* | *SKAP1* | *ATP1A1* | *NECAP2* | *GZMH* | *ENO1* | *IL32* |
| *FAM96B* | *UBE2V1* | *P2RY8* | *HIST1H4I* | *CX3CR1* | *ABHD17A* | *PKM* | *GNLY* |
| *CCDC88C* | *CTSH* | *CPVL* | *LIME1* | *DENND2D* | *ARF5* | *PRF1* | *LYZ* |
| *TATDN2* | *CHD3* | *SH3BGRL* | *TPST2* | *XBP1* | *LINC00861* | *EVL* | *FTH1* |
| *CSTB* | *CCR1* | *S1PR1* | *RUNX3* | *LPCAT1* | *ZAP70* | *AHNAK* |  |
