# Supplementary material for: Identification of aberrant innate and adaptive immunity based on changes in global gene expression in the blood of adults with autism spectrum disorder
Source: J Neuroinflammation. 2021 Apr 30;18:102. doi: 10.1186/s12974-021-02154-7 (PMC8086363; doi:10.1186/s12974-021-02154-7)
Supplement: Supplementary file 8 — Additional file 8: Table S5. Results of gene ontology for all 479 genes included in the MEbrown4 module. [file 12974_2021_2154_MOESM8_ESM.docx]

| GOID | GO Term | Benjamini-Hochberg  P value | Number of genes |
| --- | --- | --- | --- |
| **Biological process** | | | |
| GO:0021997 | neural plate axis specification | 0.01630 | 2 |
| GO:0030204 | chondroitin sulfate metabolic process | 0.04460 | 4 |
| GO:0030834 | regulation of actin filament depolymerization | 0.04231 | 5 |
| GO:0031529 | ruffle organization | 0.04148 | 5 |
| GO:0032074 | negative regulation of nuclease activity | 0.04134 | 2 |
| GO:0038036 | sphingosine-1-phosphate receptor activity | 0.04459 | 2 |
| GO:0046519 | sphingoid metabolic process | 0.04397 | 3 |
| GO:0007212 | dopamine receptor signaling pathway | 0.02045 | 6 |
| GO:0048541 | Peyer's patch development | 0.04459 | 2 |
| GO:0061817 | endoplasmic reticulum-plasma membrane tethering | 0.04180 | 2 |
| GO:1902004 | positive regulation of amyloid-beta formation | 0.04391 | 3 |
| GO:1903721 | positive regulation of I-kappaB phosphorylation | 0.04134 | 2 |
| GO:1904018 | positive regulation of vasculature development | 0.04013 | 11 |
| GO:0008277 | regulation of G protein-coupled receptor signaling pathway | 0.03026 | 10 |
| GO:0003382 | epithelial cell morphogenesis | 0.01334 | 6 |
| GO:0008310 | single-stranded DNA 3'-5' exodeoxyribonuclease activity | 0.04180 | 2 |
| GO:0015491 | cation:cation antiporter activity | 0.04047 | 4 |
| GO:0016558 | protein import into peroxisome matrix | 0.03083 | 3 |
| GO:0017183 | peptidyl-diphthamide biosynthetic process from peptidyl-histidine | 0.04459 | 2 |
| GO:0019240 | citrulline biosynthetic process | 0.04459 | 2 |
| GO:0021796 | cerebral cortex regionalization | 0.04134 | 2 |
| GO:0004715 | non-membrane spanning protein tyrosine kinase activity | 0.03219 | 6 |
| GO:0004713 | protein tyrosine kinase activity | 0.03253 | 14 |
| GO:0015918 | sterol transport | 0.03293 | 8 |
| GO:0033344 | cholesterol efflux | 0.03565 | 5 |
| GO:0008347 | glial cell migration | 0.04358 | 5 |
| GO:0043615 | astrocyte cell migration | 0.04459 | 2 |
| GO:0009225 | nucleotide-sugar metabolic process | 0.04449 | 4 |
| GO:0006258 | UDP-glucose catabolic process | 0.01630 | 2 |
| GO:0045719 | negative regulation of glycogen biosynthetic process | 0.04134 | 2 |
| GO:0045732 | positive regulation of protein catabolic process | 0.04535 | 12 |
| GO:1903364 | positive regulation of cellular protein catabolic process | 0.03178 | 10 |
| GO:1903052 | positive regulation of proteolysis involved in cellular protein catabolic process | 0.04409 | 8 |
| GO:1990034 | calcium ion export across plasma membrane | 0.04459 | 2 |
| GO:0099509 | regulation of presynaptic cytosolic calcium ion concentration | 0.04397 | 3 |
| GO:0060401 | cytosolic calcium ion transport | 0.04125 | 11 |
| GO:1901660 | calcium ion export | 0.01502 | 4 |
| GO:0097553 | calcium ion transmembrane import into cytosol | 0.04418 | 9 |
| GO:0010822 | positive regulation of mitochondrion organization | 0.04207 | 8 |
| GO:1903749 | positive regulation of establishment of protein localization to mitochondrion | 0.01603 | 7 |
| GO:2001235 | positive regulation of apoptotic signaling pathway | 0.03181 | 11 |
| GO:0097345 | mitochondrial outer membrane permeabilization | 0.02597 | 6 |
| GO:1901030 | positive regulation of mitochondrial outer membrane permeabilization involved in apoptotic signaling pathway | 0.01718 | 6 |
| GO:0003206 | cardiac chamber morphogenesis | 0.04707 | 8 |
| GO:0055017 | cardiac muscle tissue growth | 0.02307 | 8 |
| GO:0003231 | cardiac ventricle development | 0.04494 | 8 |
| GO:0003281 | ventricular septum development | 0.04054 | 6 |
| GO:0003150 | muscular septum morphogenesis | 0.03459 | 2 |
| GO:0072676 | lymphocyte migration | 0.04089 | 8 |
| GO:0046631 | alpha-beta T cell activation | 0.01017 | 12 |
| GO:0002724 | regulation of T cell cytokine production | 0.04046 | 4 |
| GO:0030098 | lymphocyte differentiation | 0.02222 | 19 |
| GO:0036037 | CD8-positive, alpha-beta T cell activation | 0.03311 | 4 |
| GO:1902105 | regulation of leukocyte differentiation | 0.03337 | 15 |
| GO:1902107 | positive regulation of leukocyte differentiation | 0.04337 | 9 |
| GO:0045058 | T cell selection | 0.02027 | 6 |
| GO:0030217 | T cell differentiation | 0.03541 | 14 |
| GO:0045619 | regulation of lymphocyte differentiation | 0.01954 | 12 |
| GO:0045577 | regulation of B cell differentiation | 0.03240 | 4 |
| GO:0045580 | regulation of T cell differentiation | 0.03988 | 9 |
| GO:0046632 | alpha-beta T cell differentiation | 0.02105 | 9 |
| GO:0043373 | CD4-positive, alpha-beta T cell lineage commitment | 0.04042 | 3 |
| GO:0002702 | positive regulation of production of molecular mediator of immune response | 0.03828 | 8 |
| GO:0072676 | lymphocyte migration | 0.04089 | 8 |
| GO:0002637 | regulation of immunoglobulin production | 0.04221 | 6 |
| GO:0002821 | positive regulation of adaptive immune response | 0.04234 | 8 |
| GO:0002706 | regulation of lymphocyte mediated immunity | 0.04348 | 9 |
| GO:0004896 | cytokine receptor activity | 0.04025 | 7 |
| GO:0019722 | calcium-mediated signaling | 0.04109 | 13 |
| GO:0033634 | positive regulation of cell-cell adhesion mediated by integrin | 0.04180 | 2 |
| GO:0070098 | chemokine-mediated signaling pathway | 0.03426 | 7 |
| GO:0002724 | regulation of T cell cytokine production | 0.04046 | 4 |
| GO:1901623 | regulation of lymphocyte chemotaxis | 0.03041 | 4 |
| GO:0004950 | chemokine receptor activity | 0.01259 | 5 |
| GO:0090026 | positive regulation of monocyte chemotaxis | 0.04397 | 3 |
| GO:2000501 | regulation of natural killer cell chemotaxis | 0.04459 | 2 |
| GO:0010820 | positive regulation of T cell chemotaxis | 0.03477 | 3 |
| GO:0045580 | regulation of T cell differentiation | 0.03988 | 9 |
| **Cellular components** | | | |
| none | | | |
| **Molecular functions** | | | |
| GO:0001046 | core promoter sequence-specific DNA binding | 0.02188 | 5 |
| GO:1905097 | regulation of guanyl-nucleotide exchange factor activity | 0.04800 | 2 |
| GO:0004950 | chemokine receptor activity | 0.01132 | 5 |
| GO:0007175 | negative regulation of epidermal growth factor-activated receptor activity | 0.02378 | 3 |
| GO:0008310 | single-stranded DNA 3'-5' exodeoxyribonuclease activity | 0.02883 | 2 |
| GO:0015491 | cation:cation antiporter activity | 0.02978 | 4 |
| GO:1905056 | calcium-transporting ATPase activity involved in regulation of presynaptic cytosolic calcium ion concentration | 0.03175 | 2 |
| GO:0004629 | phospholipase C activity | 0.02912 | 6 |
| GO:0004435 | phosphatidylinositol phospholipase C activity | 0.02607 | 4 |
| GO:0004713 | protein tyrosine kinase activity | 0.02246 | 14 |
| GO:0004715 | non-membrane spanning protein tyrosine kinase activity | 0.04678 | 6 |
