# Supplementary material for: Identification of aberrant innate and adaptive immunity based on changes in global gene expression in the blood of adults with autism spectrum disorder
Source: J Neuroinflammation. 2021 Apr 30;18:102. doi: 10.1186/s12974-021-02154-7 (PMC8086363; doi:10.1186/s12974-021-02154-7)
Supplement: Supplementary file 9 — Additional file 9: Table S6. Correlation of gene expression with age and sex. [file 12974_2021_2154_MOESM9_ESM.docx]

**Table S6. Correlation of gene expression with age and sex**

| **Variables** | **Group** | ***MAFB*** | ***MARCKS3*** | ***ALDH2*** | ***ETV7*** | ***BATF2*** | ***GNLY*** | ***SCARNA1*** | ***CROCCP2*** |
| --- | --- | --- | --- | --- | --- | --- | --- | --- | --- |
| Age | ASD | P = 0.141  r = 0.361 | P = 0.252  r = 0.285 | **P = 0.019**  **r = 0.534** | P = 0.302  r = -0.266 | P = 0.536  r = -0.161 | P = 0.777  r = 0.070 | P = 0.453  r = 0.183 | P = 0.294  r = 0.254 |
|  | Ct | P = 0.351  r = 0.227 | P = 0.416  r = 0.204 | P = 0.635  r = 0.120 | P = 0.302  r = 0.257 | P = 0.693  r = -0.097 | P = 0.288  r = 0.265 | P = 0.272  r = 0.265 | P = 0.150  r = 0.343 |
| Sex | ASD | P = 0.073 | P = 0.275 | P = 0.073 | P = 0.925 | P = 0.808 | P = 0.540 | P = 0.088 | P = 0.261 |
|  | Ct | P = 0.988 | P = 0.402 | P = 0.319 | P = 0.092 | P = 0.257 | P = 0.446 | P = 0.182 | P = 0.540 |

| **Variables** | **Group** | ***RPSAP58*** | ***G2MK*** | ***TLR1*** | ***IL1B*** | ***TNFAIP6*** |
| --- | --- | --- | --- | --- | --- | --- |
| Age | ASD | P = 0.379  r = -0.214 | P = 0.248  r = -0.278 | P = 0.523  r = 0.156 | P = 0.357  r = 0.231 | P = 0.363  r = -0.235 |
|  | Ct | P = 0.539  r = 0.150 | P = 0.267  r = 0.268 | P = 0.190  r = -0.314 | P = 0.540  r = 0.155 | P = 0.114  r = 0.375 |
| Sex | ASD | **P = 0.031** | P = 0.643 | P = 0.482 | P = 0.593 | **P = 0.008** |
|  | Ct | P = 0.196 | P = 0.449 | P = 0.889 | P = 0.734 | P = 0.833 |
